# Supplementary material for: The Antitumor Effect of the DNA Polymerase Alpha Inhibitor ST1926 in Glioblastoma: A Proteomics Approach
Source: Int J Mol Sci. 2023 Sep 14;24(18):14069. doi: 10.3390/ijms241814069 (PMC10531065; doi:10.3390/ijms241814069)
Supplement: Supplementary file 1 [file ijms-24-14069-s001.zip › Revised Supplementary Figures.pptx]

## Slide 1
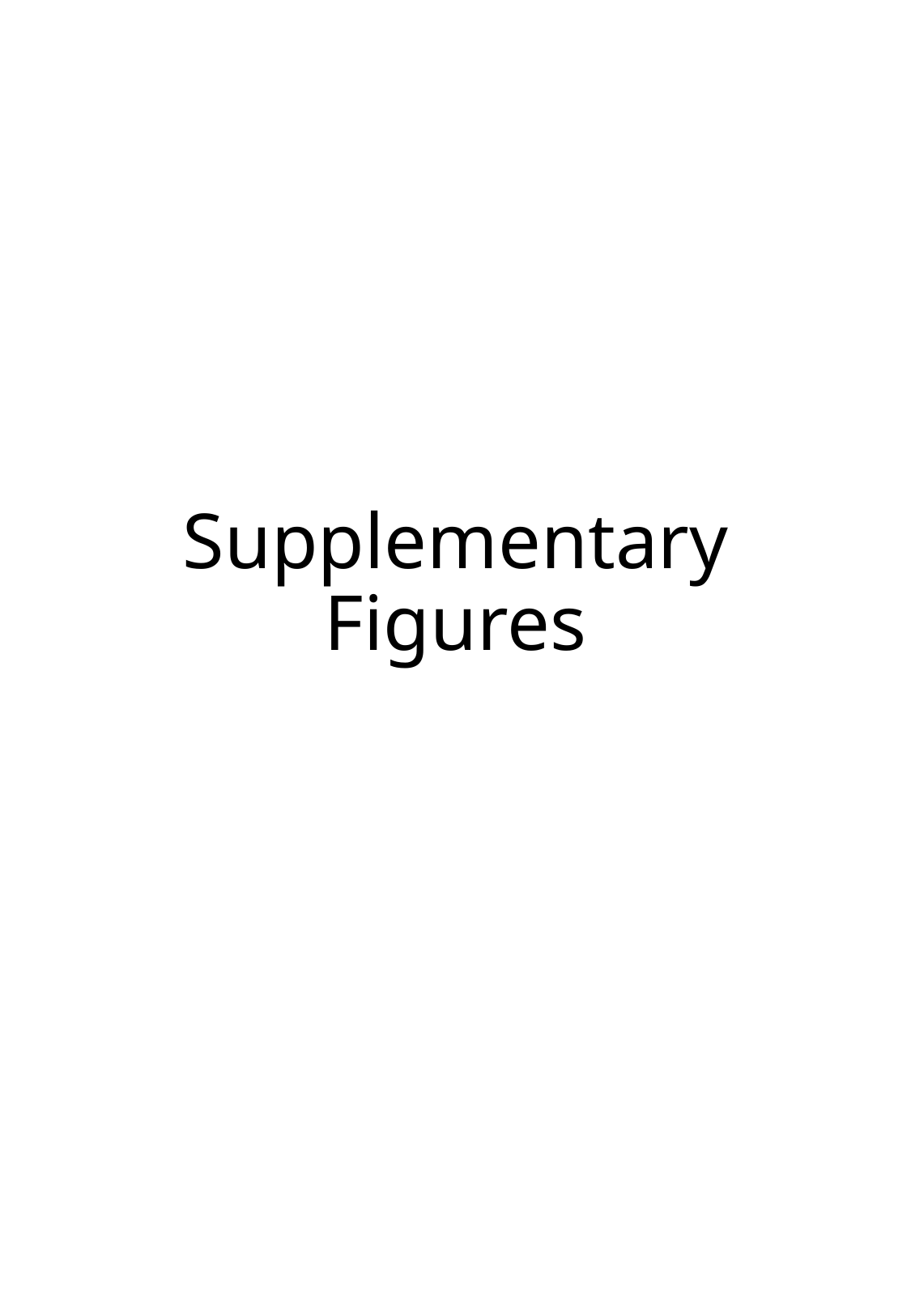

# Supplementary Figures

## Slide 2
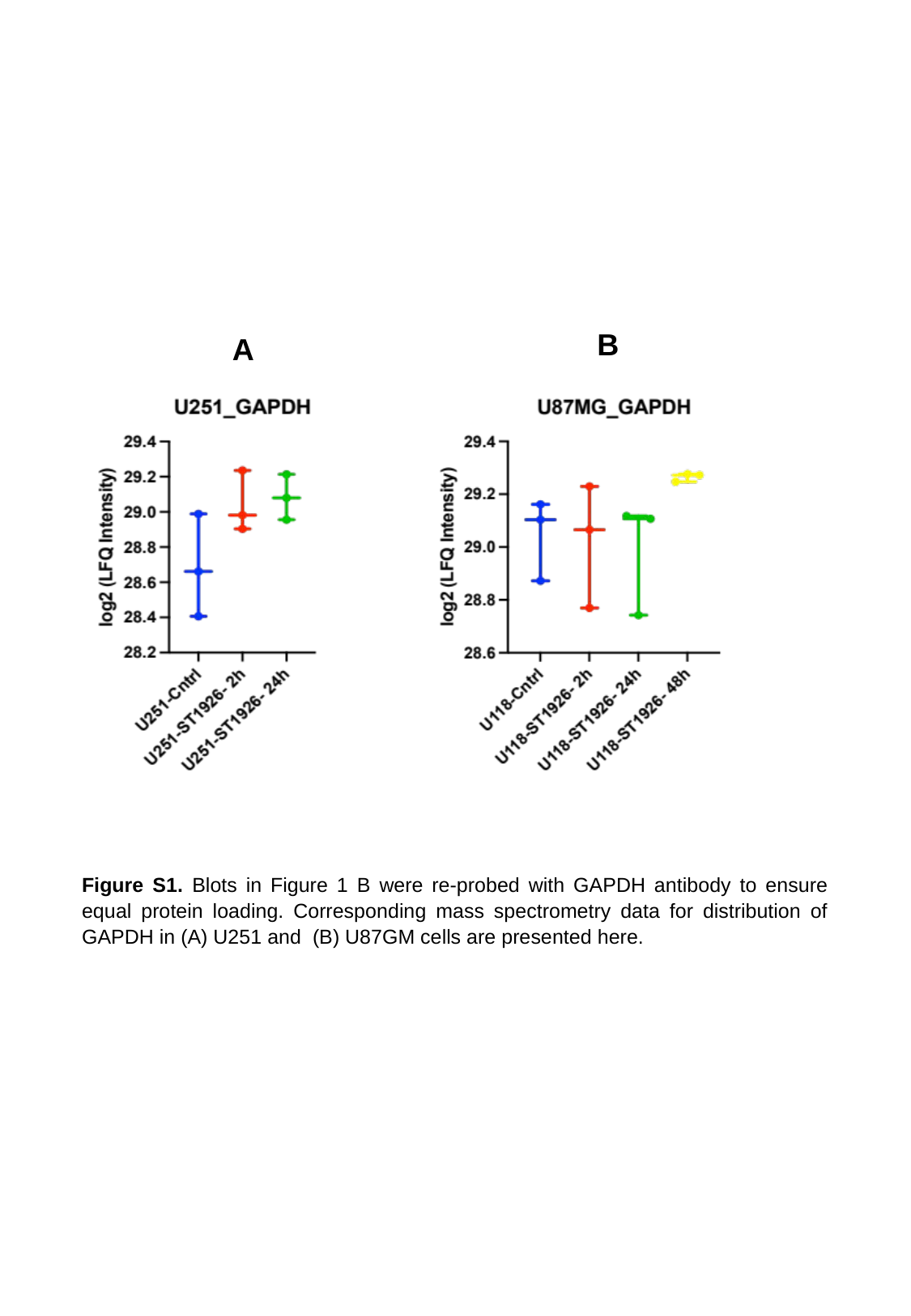

B
A
Figure S1. Blots in Figure 1 B were re-probed with GAPDH antibody to ensure equal protein loading. Corresponding mass spectrometry data for distribution of GAPDH in (A) U251 and (B) U87GM cells are presented here.

## Slide 3
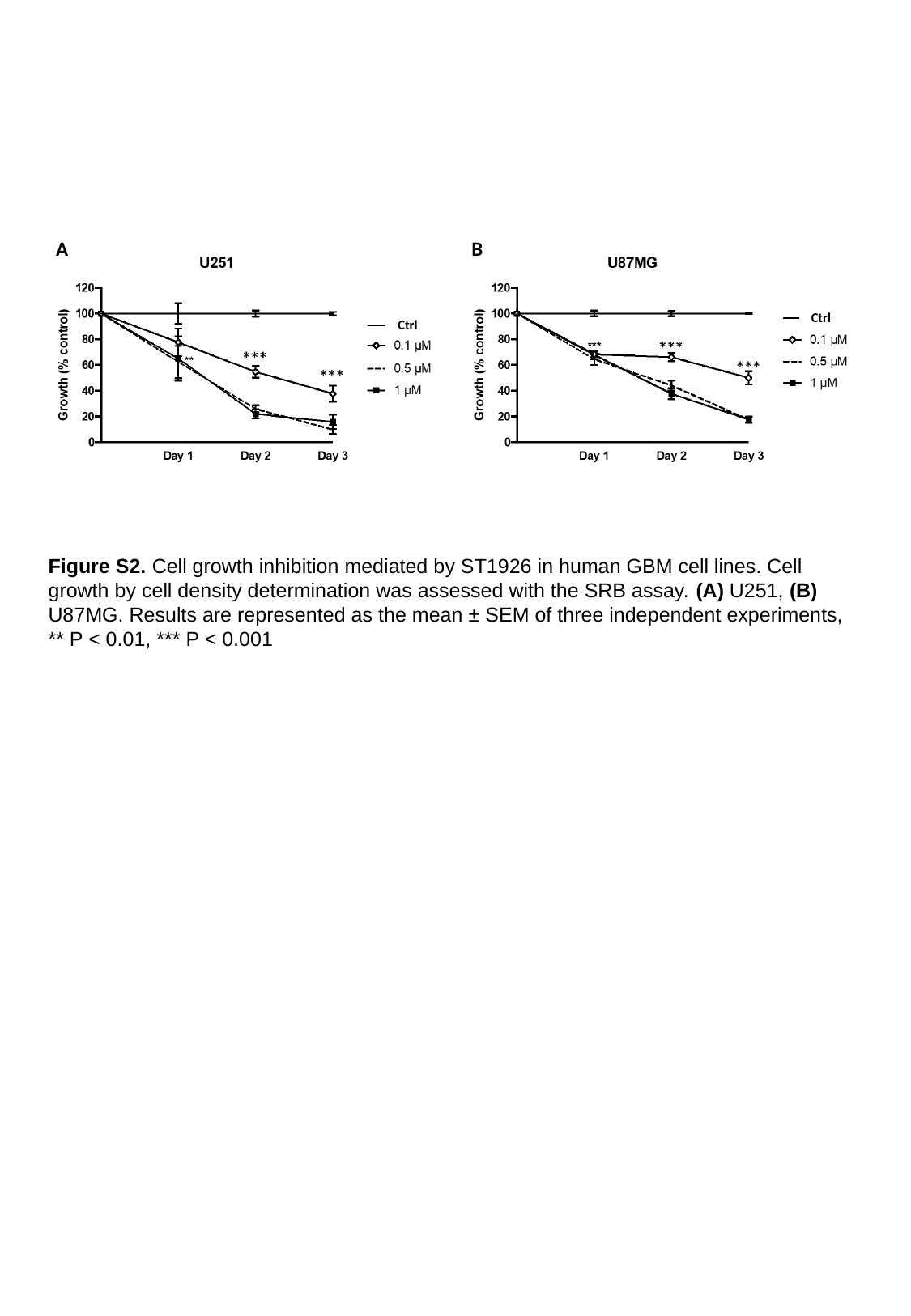

A
B
Ctrl
Ctrl
Figure S2. Cell growth inhibition mediated by ST1926 in human GBM cell lines. Cell growth by cell density determination was assessed with the SRB assay. (A) U251, (B) U87MG. Results are represented as the mean ± SEM of three independent experiments, ** P < 0.01, *** P < 0.001

## Slide 4
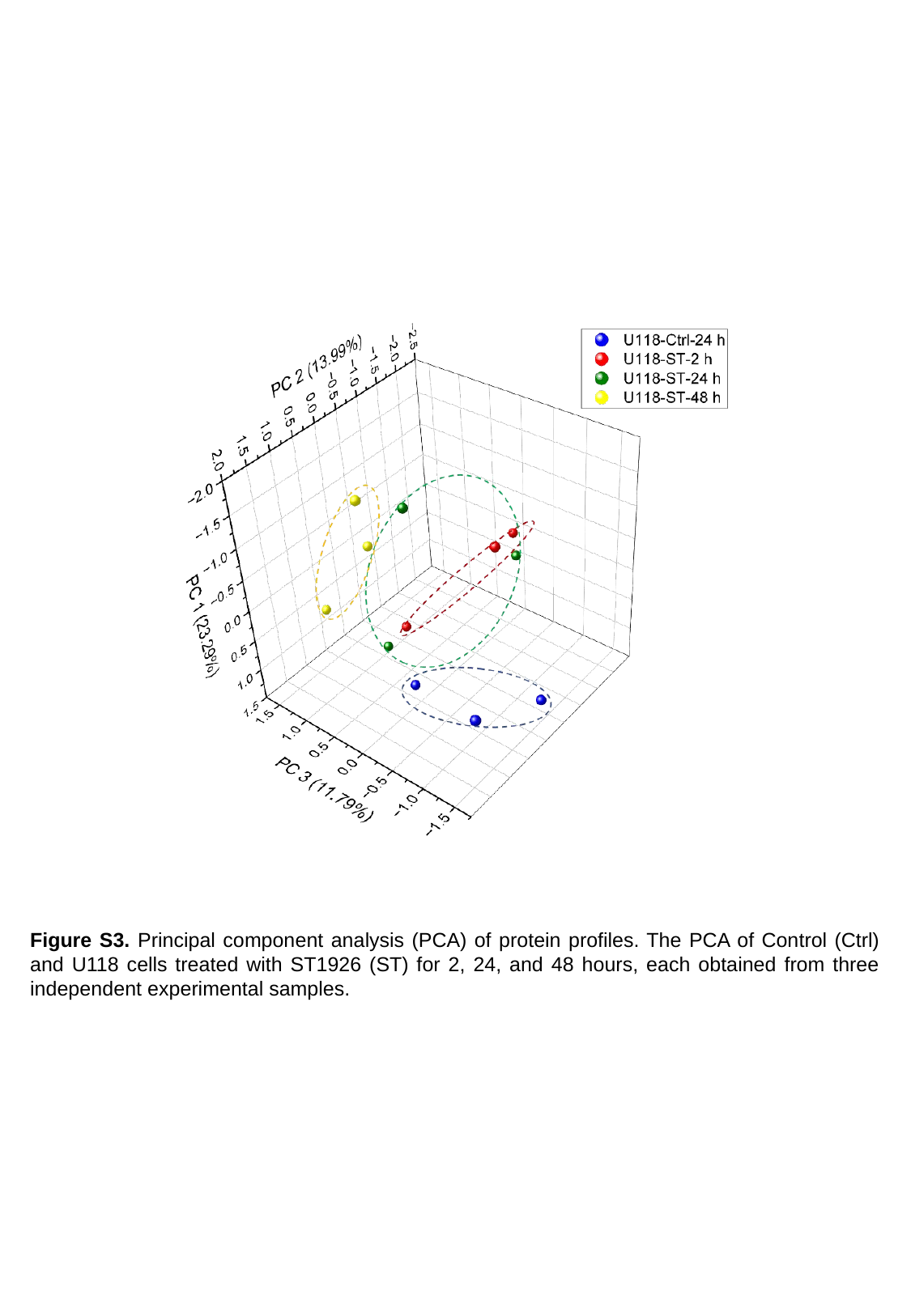

Figure S3. Principal component analysis (PCA) of protein profiles. The PCA of Control (Ctrl) and U118 cells treated with ST1926 (ST) for 2, 24, and 48 hours, each obtained from three independent experimental samples.

## Slide 5
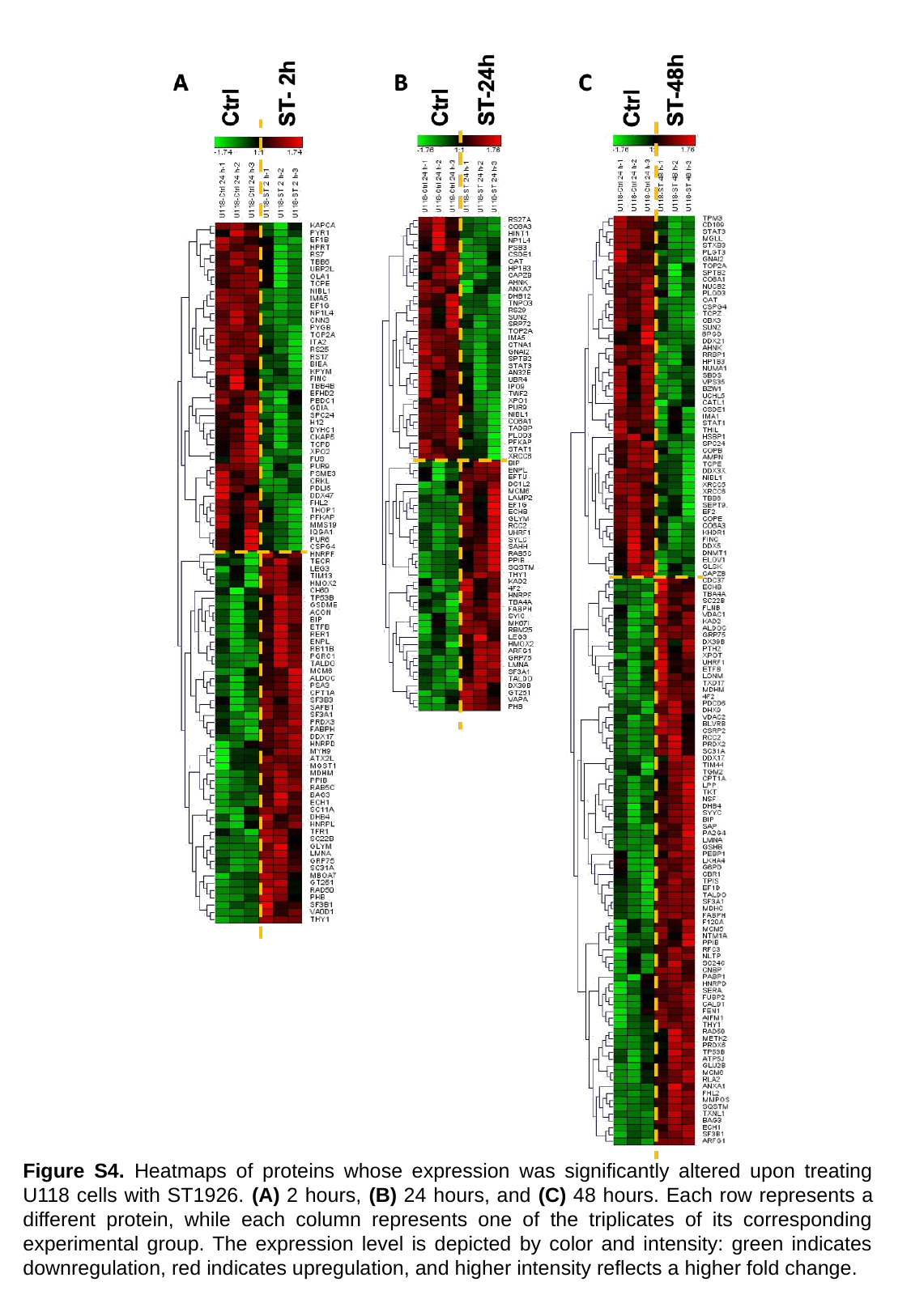

Figure S4. Heatmaps of proteins whose expression was significantly altered upon treating U118 cells with ST1926. (A) 2 hours, (B) 24 hours, and (C) 48 hours. Each row represents a different protein, while each column represents one of the triplicates of its corresponding experimental group. The expression level is depicted by color and intensity: green indicates downregulation, red indicates upregulation, and higher intensity reflects a higher fold change.

## Slide 6
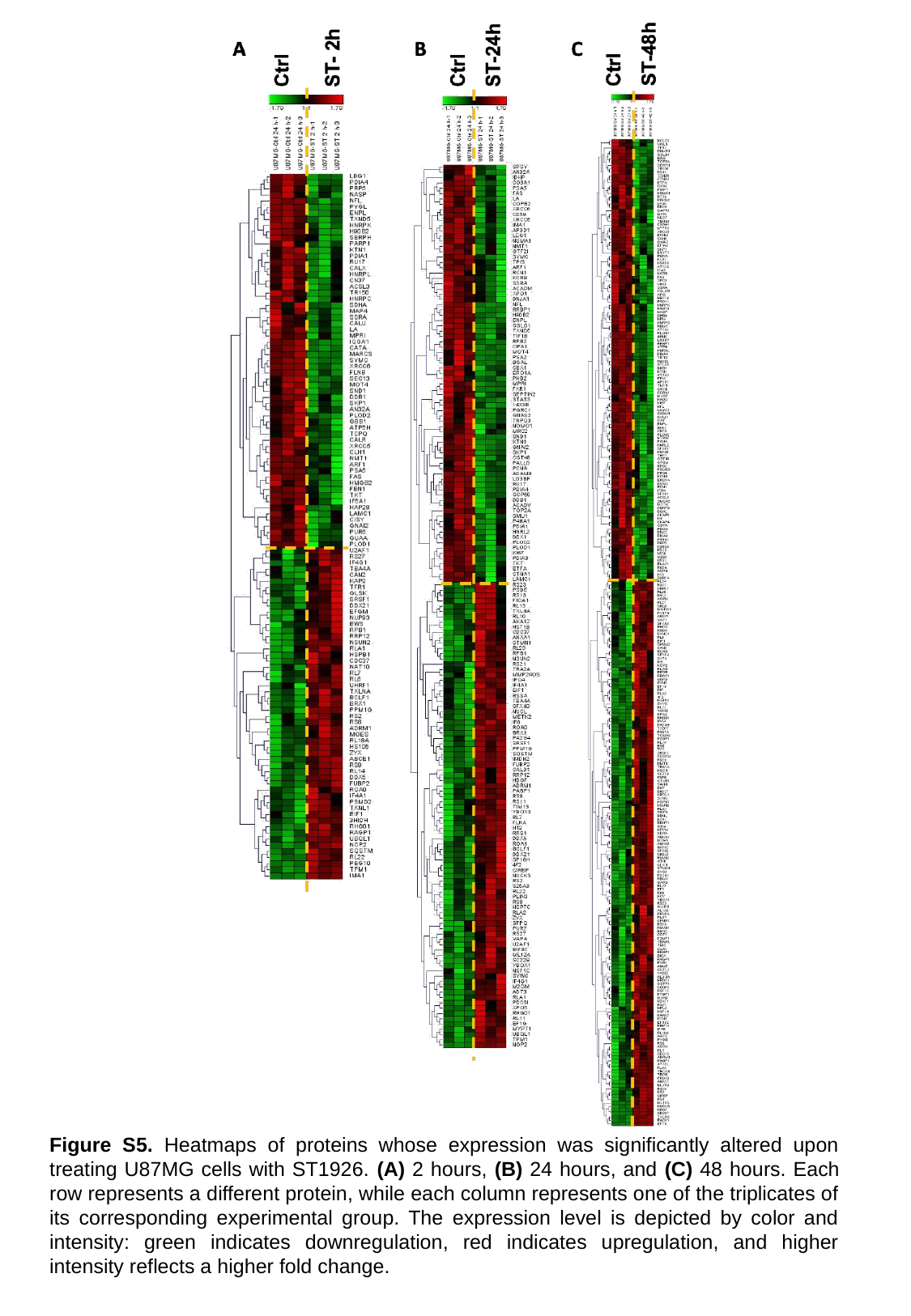

Figure S5. Heatmaps of proteins whose expression was significantly altered upon treating U87MG cells with ST1926. (A) 2 hours, (B) 24 hours, and (C) 48 hours. Each row represents a different protein, while each column represents one of the triplicates of its corresponding experimental group. The expression level is depicted by color and intensity: green indicates downregulation, red indicates upregulation, and higher intensity reflects a higher fold change.

## Slide 7
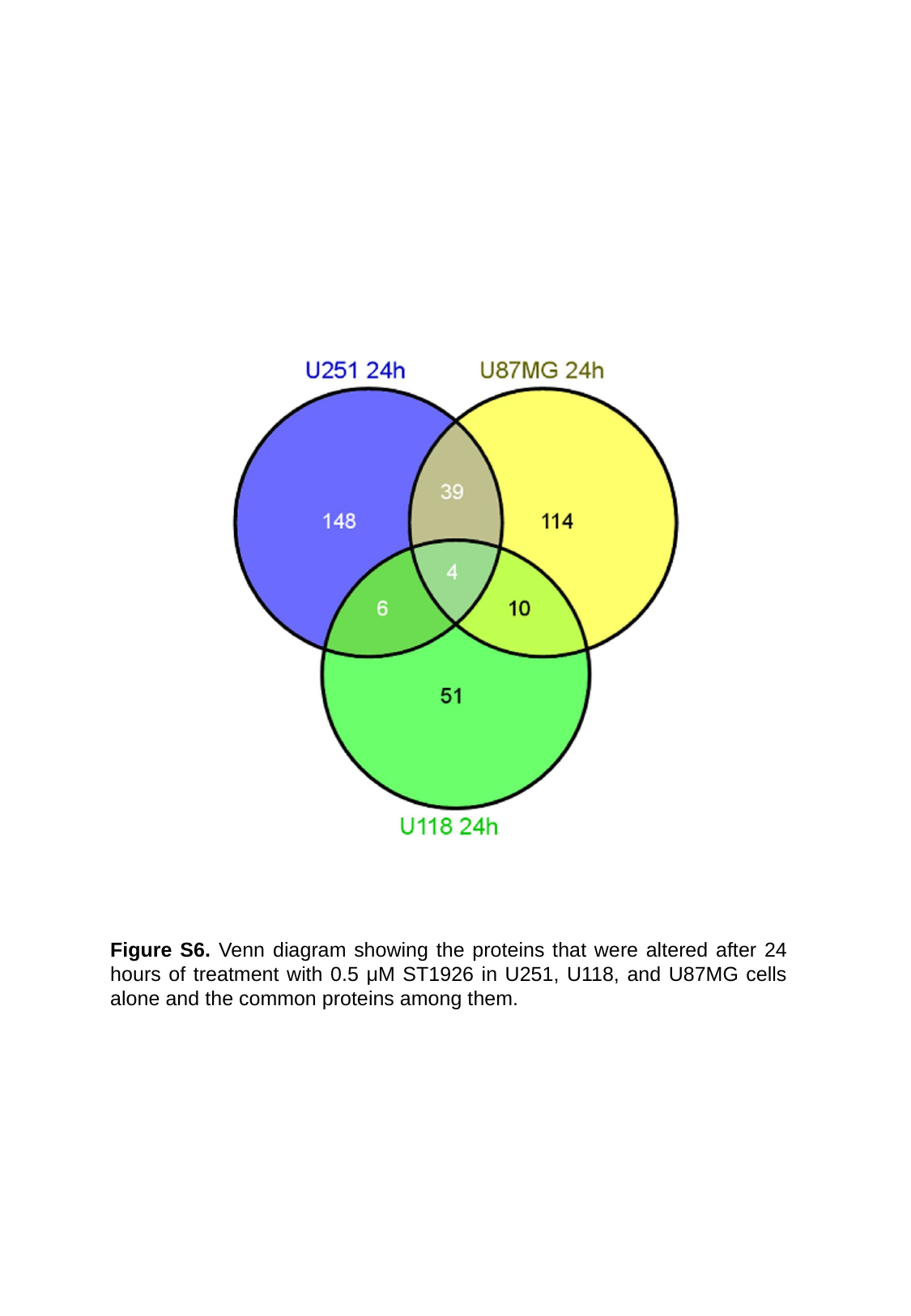

Figure S6. Venn diagram showing the proteins that were altered after 24 hours of treatment with 0.5 μM ST1926 in U251, U118, and U87MG cells alone and the common proteins among them.

## Slide 8
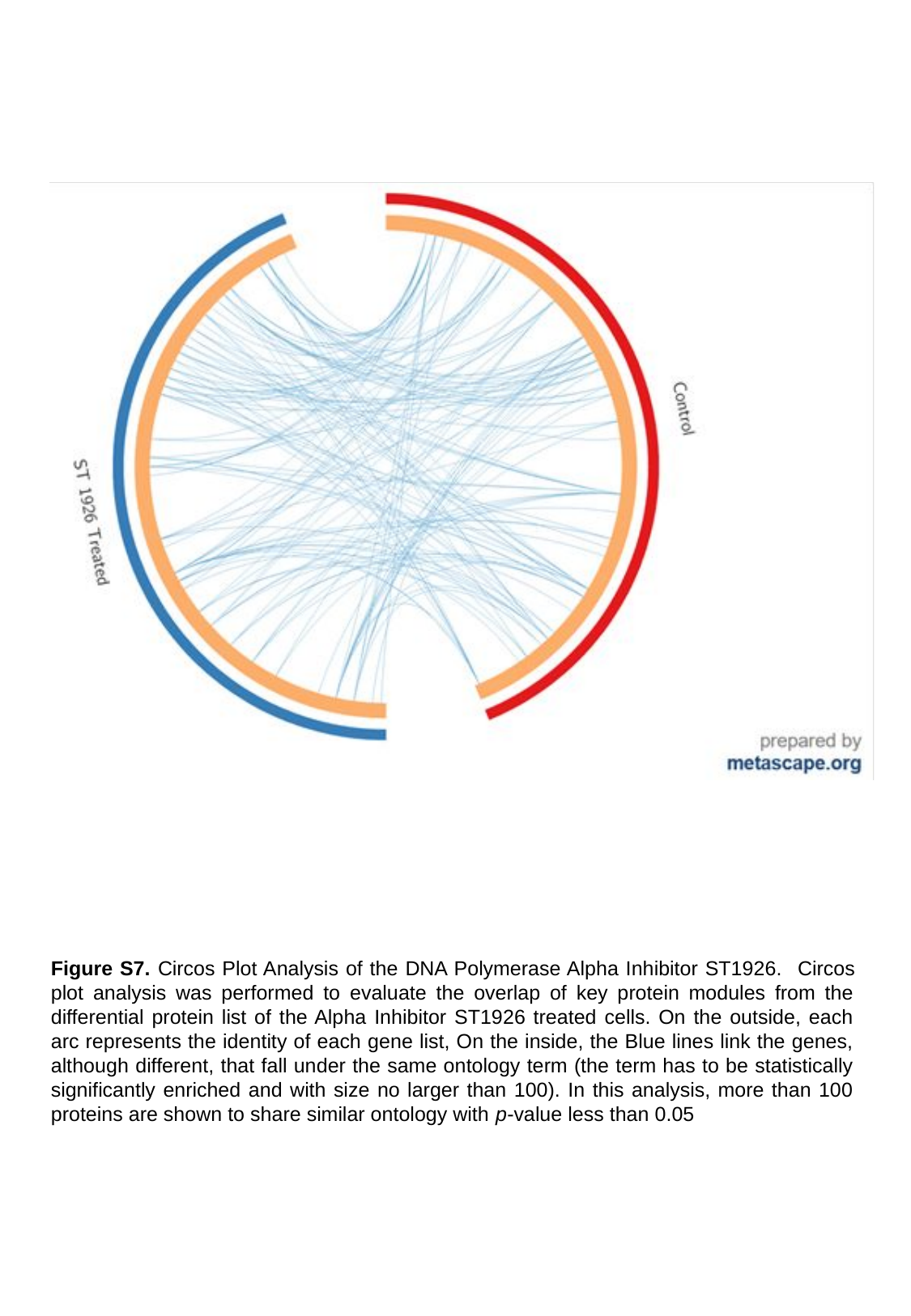

Figure S7. Circos Plot Analysis of the DNA Polymerase Alpha Inhibitor ST1926. Circos plot analysis was performed to evaluate the overlap of key protein modules from the differential protein list of the Alpha Inhibitor ST1926 treated cells. On the outside, each arc represents the identity of each gene list, On the inside, the Blue lines link the genes, although different, that fall under the same ontology term (the term has to be statistically significantly enriched and with size no larger than 100). In this analysis, more than 100 proteins are shown to share similar ontology with p-value less than 0.05

## Slide 9
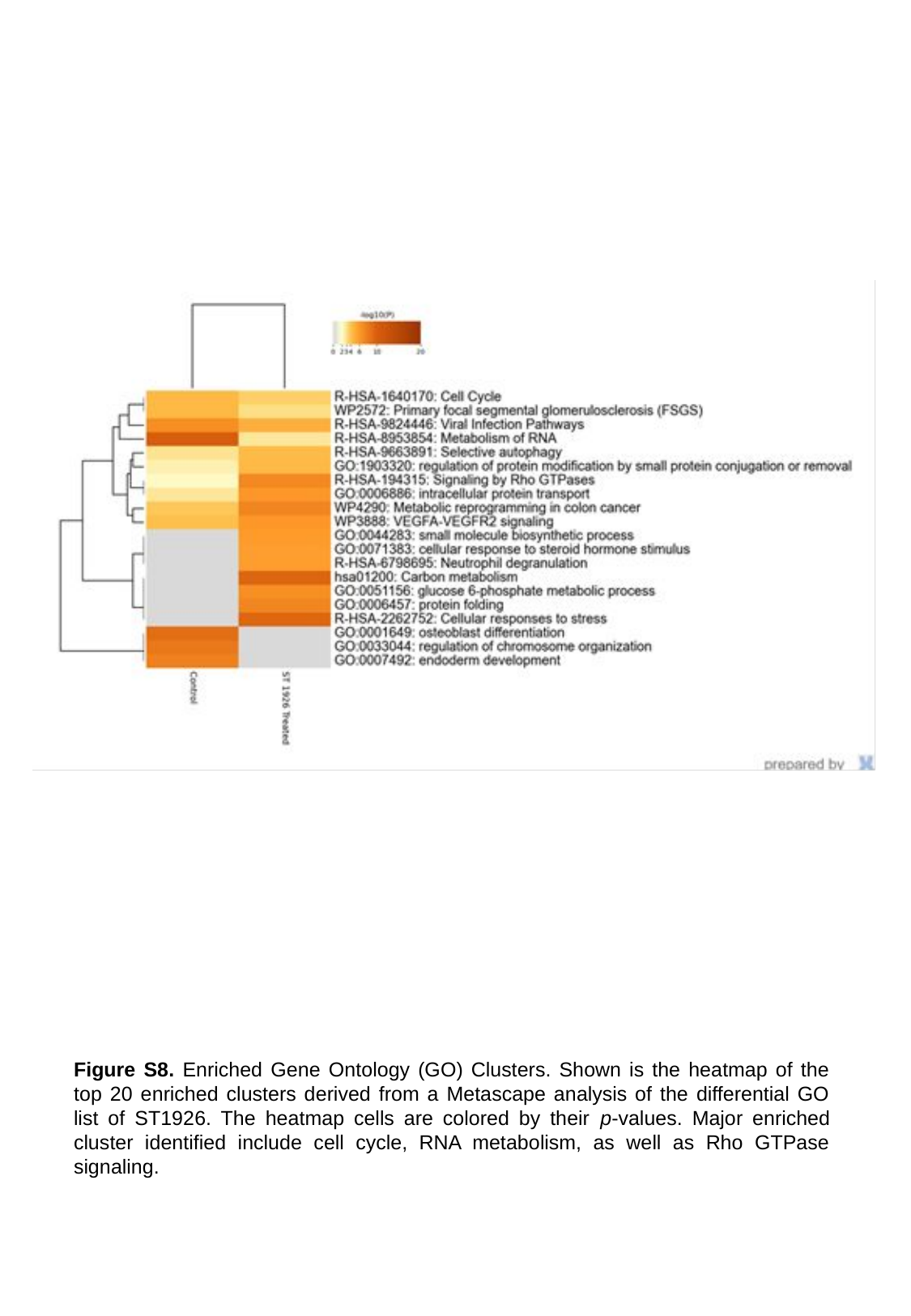

Figure S8. Enriched Gene Ontology (GO) Clusters. Shown is the heatmap of the top 20 enriched clusters derived from a Metascape analysis of the differential GO list of ST1926. The heatmap cells are colored by their p-values. Major enriched cluster identified include cell cycle, RNA metabolism, as well as Rho GTPase signaling.

## Slide 10
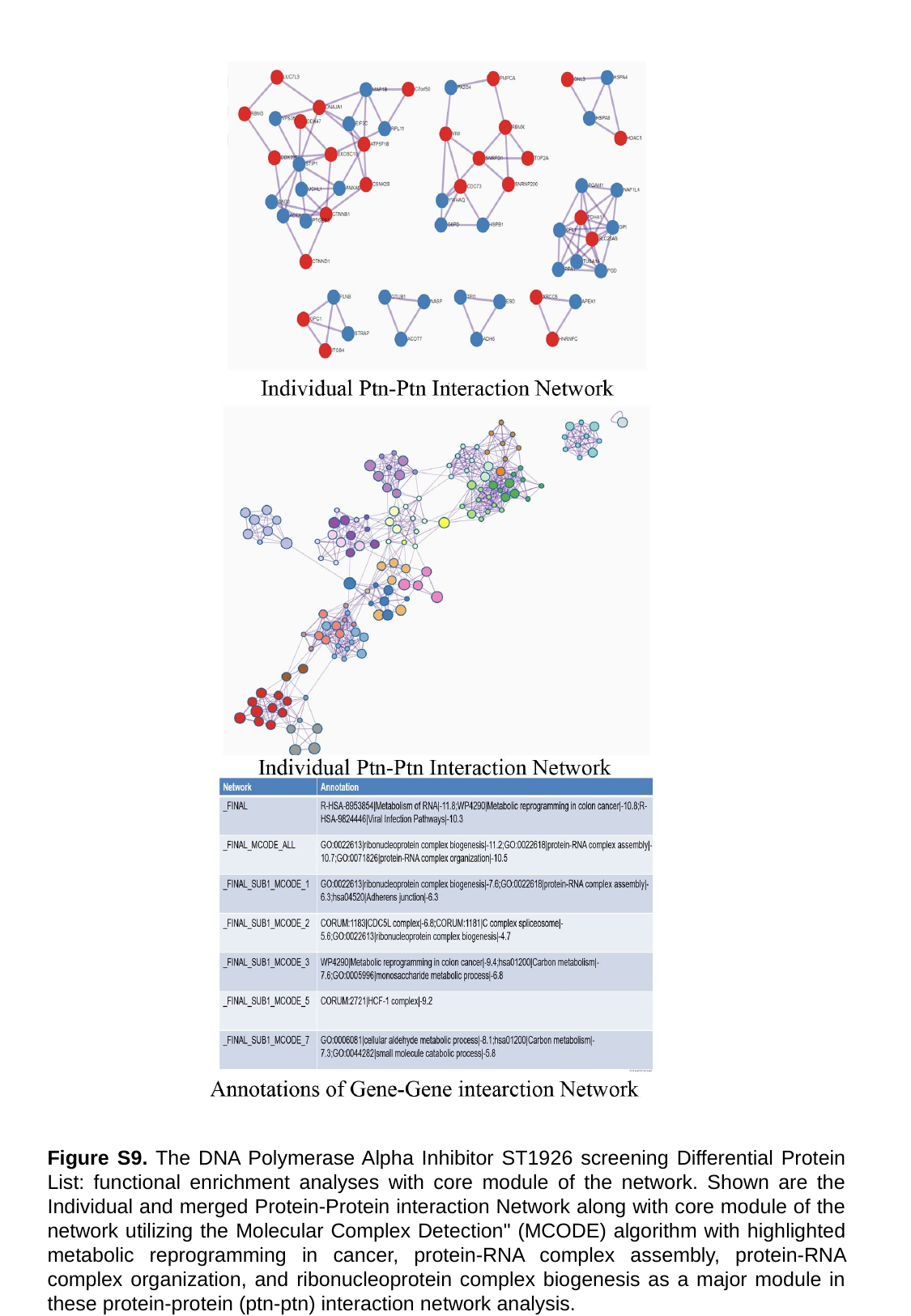

Figure S9. The DNA Polymerase Alpha Inhibitor ST1926 screening Differential Protein List: functional enrichment analyses with core module of the network. Shown are the Individual and merged Protein-Protein interaction Network along with core module of the network utilizing the Molecular Complex Detection" (MCODE) algorithm with highlighted metabolic reprogramming in cancer, protein-RNA complex assembly, protein-RNA complex organization, and ribonucleoprotein complex biogenesis as a major module in these protein-protein (ptn-ptn) interaction network analysis.
