# Supplementary material for: The Antitumor Effect of the DNA Polymerase Alpha Inhibitor ST1926 in Glioblastoma: A Proteomics Approach
Source: Int J Mol Sci. 2023 Sep 14;24(18):14069. doi: 10.3390/ijms241814069 (PMC10531065; doi:10.3390/ijms241814069)
Supplement: Supplementary file 1 [file ijms-24-14069-s001.zip › Revised Supplementary Tables.pdf]

Supplementary Tables:

Table S1. List of altered proteins in U251 cells versus Control, after treatment with 0.5 µM ST1926 for 2 hours. A total of 77 proteins with significant changes (30 downregulated (↓) and 47 upregulated (↑)) were identified in U251 cells with $p < 0.05$.

Protein IDs	Protein Names	Gene IDs	*p*-value	Fold Change
Q14980	Nuclear mitotic apparatus protein 1	NUMA1	0.0007	0.99 ↓
Q99714	3-hydroxyacyl-CoA dehydrogenase type-2 (HCD2)	HSD17B10	0.0008	0.98 ↓
P00387	NADH-cytochrome b5 reductase 3	CYB5R3	0.0009	0.98 ↓
Q9C0H2	Protein tweety homolog 3	TTYH3	0.002	0.97 ↓
P35659	Protein DEK	DEK	0.005	0.98 ↓
P07237	Protein disulfide-isomerase	PDI	0.005	0.99 ↓
P07954	Fumarate hydratase	FH	0.006	0.97 ↓
O14979	Heterogeneous nuclear ribonucleoprotein D-like	HNRNPDL	0.007	0.99 ↓
Q9Y277	Voltage-dependent anion-selective channel protein 3	VDAC-3	0.008	0.98 ↓
O00159	Unconventional myosin-Ic	MYO1C	0.01	0.98 ↓
P50454	Serpin family H member 1	SERPINH1	0.01	0.98 ↓
Q9Y2W1	Thyroid hormone receptor-associated protein 3	THRAP3	0.01	0.98 ↓
Q13084	39S ribosomal protein L28	MRP-L28	0.02	0.98 ↓
P61026	Ras-related protein Rab-10	RAB10	0.02	0.99 ↓
Q14257	Reticulocalbin-2/ (E6-binding protein) (E6BP)	RCN2	0.02	0.99 ↓
P26599	Polypyrimidine tract-binding protein 1	PTBP1	0.02	0.99 ↓
Q13838	Spliceosome RNA helicase DDX39B	DDX39B	0.02	0.98 ↓
Q86UP2	Kinectin	KTN1	0.03	0.99 ↓
Q9UH99	SUN domain-containing protein 2	SUN2	0.03	0.98 ↓
P27797	Calreticulin (CRP55) (Calregulin)	CALR	0.03	0.99 ↓
Q9H857	5'-nucleotidase domain-containing protein 2	NT5DC2	0.03	0.97 ↓
Q9Y4L1	Hypoxia up-regulated protein 1	HYOU1	0.03	0.99 ↓
P01111	GTPase NRas	NRAS	0.03	0.97 ↓
P08621	U1 small nuclear ribonucleoprotein 70 kDa	SNRNP70	0.04	0.98 ↓
P11387	DNA topoisomerase 1	TOP1	0.05	0.97 ↓
P12236	Solute carrier family 25 member 6	SLC25A6	0.04	0.98 ↓
P11166	Solute carrier family 2, facilitated glucose transporter member 1	SLC2A1 GLUT1	0.04	0.94 ↓
Q12906	Interleukin enhancer-binding factor 3	ILF3	0.04	0.99 ↓
Q13185	Chromobox protein homolog 3	CBX3	0.04	0.98 ↓
Q9BRJ6	Uncharacterized protein C7orf50	C7orf50	0.04	0.99 ↓

Protein IDs	Protein Names	Gene IDs	*p*-value	Fold Change
O00299	Chloride intracellular channel protein 1	CLIC1	0.02	1.02 ↑
Q9BZQ8	Protein Niban 1 (Protein FAM129A)	NIBAN1 FAM129A	0.05	1.02 ↑
Q9BVA1	Tubulin beta-2B chain	TUBB2B	0.04	1.03 ↑
Q13620	Cullin-4B (CUL-4B)	CUL4B	0.04	1.01 ↑
P15151	Poliovirus receptor	PVR/ PVS	0.04	1.03 ↑
P27694	Replication protein A 70 kDa DNA-binding subunit	RPA1 RPA70	0.04	1.02 ↑
P49327	Fatty acid synthase	FASN/ FAS	0.04	1.01 ↑
P06737	Glycogen phosphorylase	PYGL	0.04	1.02 ↑
P32119	Peroxiredoxin-2	PRDX2	0.04	1.01 ↑
Q9BUF5	Tubulin beta-6 chain	TUBB6	0.04	1.02 ↑
P41250	Glycine--tRNA ligase	GARS1 GARS	0.04	1.01 ↑
P60174	Triosephosphate isomerase (TIM)	TPI1/ TPI	0.04	1.02 ↑
Q8TAT6	Nuclear protein localization protein 4 homolog	NPLOC4	0.04	1.03 ↑
P14618	Pyruvate kinase PKM	PKM/ OIP3	0.03	1.01 ↑
Q9NTK5	Obg-like ATPase 1	OLA1	0.03	1.01 ↑
Q15185	Prostaglandin E synthase 3	PTGES3	0.03	1.01 ↑
P30086	Phosphatidylethanolamine-binding protein 1 (PEBP-1)	PEBP1/ PBP/ PEBP	0.03	1.03 ↑
Q9Y2J8	Protein-arginine deiminase type-2	PADI2	0.03	1.02 ↑
P30085	UMP-CMP kinase	CMPK1 CMK CMPK	0.03	1.03 ↑
Q16881	Thioredoxin Reductase 1	TXNRD1	0.03	1.03 ↑
O43175	D-3-phosphoglycerate dehydrogenase (3-PGDH)	PHGDH PGDH3	0.03	1.02 ↑
Q9NZL4	Hsp70-binding protein 1 (HspBP1)	HSPBP1	0.03	1.01 ↑
P23526	Adenosylhomocysteinase (AdoHcyase)	AHCY	0.02	1.02 ↑
P31689	DnaJ homolog subfamily A member 1 (DnaJ1)	DNAJA1 DNAJ2	0.02	1.01 ↑
P52292	Importin subunit alpha-1	KPNA2	0.02	1.02 ↑
P07195	L-lactate dehydrogenase B chain (LDH-B)	LDHB	0.02	1.02 ↑
P52565	Rho GDP-dissociation inhibitor 1 (Rho GDI 1)	ARHGDIA	0.02	1.03 ↑
P06733	Alpha-enolase	ENO1	0.02	1.02 ↑
Q15102	Platelet-activating factor acetylhydrolase IB subunit alpha1	PAFAH1B3	0.02	1.02 ↑
Q14008	Cytoskeleton-associated protein 5	CKAP5	0.02	1.02 ↑

Protein IDs	Protein Names	Gene IDs	*p*-value	Fold Change
P00492	Hypoxanthine-guanine phosphoribosyltransferase (HGPRT)	HPRT1<br>HPRT	0.02	1.01 ↑
Q16658	Fascin	FSCN1	0.01	1.02 ↑
P60866	40S ribosomal protein S20	RPS20	0.01	1.03 ↑
O60884	DnaJ homolog subfamily A member 2	DNAJA2	0.01	1.01 ↑
Q9BRA2	Thioredoxin domain-containing protein 17	TXNDC17	0.01	1.04 ↑
P15559	NAD(P)H dehydrogenase [quinone] 1	NQO1	0.01	1.04 ↑
O75083	WD repeat-containing protein 1	WDR1	0.01	1.03 ↑
Q99497	Parkinson disease protein 7	PARK7	0.01	1.02 ↑
Q9H223	EH domain-containing protein 4	EHD4	0.01	1.02 ↑
P31150	Rab GDP dissociation inhibitor alpha (Rab GDI alpha)	GDI1<br>GDIL	0.01	1.03 ↑
P06744	Glucose-6-phosphate isomerase	GPI	0.006	1.01 ↑
P04083	Annexin A1 (Annexin I)	ANXA1	0.006	1.02 ↑
P63104	14-3-3 protein zeta/delta (Protein kinase C inhibitor protein 1) (KCIP-1)	YWHAZ	0.006	1.02 ↑
O60664	Perilipin-3	PLIN3	0.003	1.03 ↑
P11586	C-1-tetrahydrofolate synthase, Methylenetetrahydrofolate dehydrogenase	MTHFD1<br>MTHFC<br>MTHFD	0.003	1.02 ↑
P08758	Annexin A5 (Annexin V)	ANXA5	0.0008	1.02 ↑
Q99613	Eukaryotic translation initiation factor 3 subunit C (eIF3c)	EIF3C<br>EIF3S8	0.0005	1.02 ↑

Table S2. List of altered proteins in U251 cells versus Control, after treatment with 0.5 µM ST1926 for 24 hours. A total of 197 proteins with significant changes (71 downregulated (↓) and 126 upregulated (↑)) were identified in U251 cells with $p < 0.05$.

Protein IDs	Protein Names	Gene IDs	*p*-value	Fold Change
Q86UP2	Kinectin	KTN1	0.0001	0.98 ↓
Q9P2E9	Ribosome-binding protein 1	RRBP1	0.0002	0.94 ↓
P11388	DNA topoisomerase 2-alpha	TOP2A TOP2	0.0004	0.92 ↓
Q9BVP2	Guanine nucleotide-binding protein-like 3	GNL3	0.0004	0.97 ↓
P52292	Importin subunit alpha-1	KPNA2	0.0005	0.96 ↓
P09486	SPARC (Secreted protein acidic and rich in cysteine)	SPARC	0.0007	0.93 ↓
P12109	Collagen alpha-1(VI) chain	COL6A1	0.0008	0.96 ↓
Q15582	Transforming growth factor-beta-induced protein ig-h3	TGFBI BIGH3	0.001	0.91 ↓
P16144	Integrin beta-4	ITGB4	0.001	0.96 ↓
P06576	ATP synthase subunit beta	ATP5F1B	0.001	0.98 ↓
P26358	DNA (cytosine-5)-methyltransferase 1 (Dnmt1)	DNMT1	0.002	0.98 ↓
P07942	Laminin subunit beta-1	LAMB1	0.002	0.95 ↓
P78347	General transcription factor II-I (GTFII-I)	GTF2I	0.002	0.98 ↓
Q01780	Exosome component 10	EXOSC10	0.003	0.96 ↓
Q13185	Chromobox protein homolog 3	CBX3	0.003	0.96 ↓
Q9Y4L1	Hypoxia up-regulated protein 1	HYOU1	0.004	0.99 ↓
Q02241	Kinesin-like protein KIF23	KIF23	0.004	0.93 ↓
P46013	Proliferation marker protein Ki-67	MKI67	0.005	0.95 ↓
Q08380	Galectin-3-binding protein (Gal-3BP)	LGALS3BP	0.006	0.96 ↓
Q8NI22	Multiple coagulation factor deficiency protein 2	MCFD2	0.006	0.97 ↓
P11166	Solute carrier family 2, facilitated glucose transporter member 1	SLC2A1 GLUT1	0.006	0.94 ↓
P16615	Sarcoplasmic/endoplasmic reticulum calcium ATPase 2 (SERCA2)	ATP2A2 ATP2B	0.006	0.98 ↓
O75643	U5 small nuclear ribonucleoprotein 200 kDa helicase	SNRNP200	0.007	0.99 ↓
O60716	Catenin delta-1 (Cadherin-associated Src substrate) (CAS)	CTNND1 KIAA0384	0.008	0.96 ↓
P05023	Sodium/potassium-transporting ATPase subunit alpha-1	ATP1A1	0.009	0.99 ↓
P31689	DnaJ homolog subfamily A member 1 (DnaJ protein homolog 2)	DNAJA1 DNAJ2	0.01	0.97 ↓
Q9H857	5'-nucleotidase domain-containing protein 2	NT5DC2	0.01	0.95 ↓

Protein IDs	Protein Names	Gene IDs	*p*-value	Fold Change
Q92896	Golgi apparatus protein 1 (CFR-1)	GLG1	0.01	0.97 ↓
P13473	Lysosome-associated membrane glycoprotein 2 (LAMP-2)	LAMP2	0.01	0.99 ↓
Q8NI27	THO complex subunit 2 (Tho2)	THOC2	0.01	0.98 ↓
Q15005	Signal peptidase complex subunit 2	SPCS2	0.01	0.96 ↓
P35222	Catenin beta-1 (Beta-catenin)	CTNNB1	0.01	0.95 ↓
Q02809	Procollagen-lysine,2-oxoglutarate 5-dioxygenase 1	PLOD1<br>LLH	0.01	0.97 ↓
P63208	S-phase kinase-associated protein 1	SKP1	0.01	0.97 ↓
P11047	Laminin subunit gamma-1 (Laminin B2 chain)	LAMB2	0.01	0.95 ↓
Q9BRJ6	Uncharacterized protein C7orf50	C7orf50	0.01	0.98 ↓
Q9BQ52	Zinc phosphodiesterase ELAC protein 2	ELAC2	0.01	0.95 ↓
Q9UH99	SUN domain-containing protein 2	SUN2	0.01	0.93 ↓
Q8TCJ2	Dolichyl-diphosphooligosaccharide--protein glycosyltransferase subunit STT3B	STT3B<br>SIMP	0.02	0.99 ↓
Q7KZF4	Staphylococcal nuclease domain-containing protein 1	SND1<br>TDRD11	0.02	0.99 ↓
Q13547	Histone deacetylase 1 (HD1)	HDAC1	0.02	0.98 ↓
Q14980	Nuclear mitotic apparatus protein 1	NUMA1	0.02	0.98 ↓
O94925	Glutaminase kidney isoform (GLS)/ (K-glutaminase)	GLS<br>GLS1	0.02	0.98 ↓
Q92922	SWI/SNF complex subunit SMARCC1	SMARCC1	0.02	0.98 ↓
P61769	Beta-2-microglobulin	B2M	0.02	0.95 ↓
P67870	Casein kinase II subunit beta (CK II beta)	CSNK2B	0.02	0.99 ↓
P35052	Glypican-1	GPC1	0.02	0.94 ↓
P63092	Guanine nucleotide-binding protein G(s) subunit alpha isoforms short	GNAS<br>GNAS1	0.02	0.97 ↓
Q6P1J9	Parafibromin	CDC73	0.02	0.93 ↓
O94826	Mitochondrial import receptor subunit TOM70	TOMM70<br>TOM70	0.02	0.99 ↓
P08670	Vimentin	VIM	0.02	0.99 ↓
P62314	Small nuclear ribonucleoprotein Sm D1	SNRPD1	0.03	0.98 ↓
Q9NSE4	Isoleucine--tRNA ligase	IARS2	0.03	0.98 ↓
P83916	Chromobox protein homolog 1 (HP1Hsbeta)	CBX1	0.03	0.97 ↓
Q9BRK5	45 kDa calcium-binding protein (Cab45)	SDF4<br>CAB45	0.03	0.98 ↓
P38159	RNA-binding motif protein, X chromosome	RBMX	0.03	0.99 ↓
Q13838	Spliceosome RNA helicase DDX39B	DDX39B	0.03	0.99 ↓
O95232	Luc7-like protein 3	LUC7L3	0.03	0.99 ↓

Protein IDs	Protein Names	Gene IDs	*p*-value	Fold Change
O43143	Pre-mRNA-splicing factor ATP-dependent RNA helicase DHX15	DHX15 DBP1	0.03	0.99 ↓
P12236	Solute carrier family 25 member 6	SLC25A6	0.04	0.98 ↓
O60832	H/ACA ribonucleoprotein complex subunit DKC1	DKC1 NOLA4	0.04	0.97 ↓
Q5SRE5	Nucleoporin NUP188 (hNup188)	NUP188	0.04	0.99 ↓
P98179	RNA-binding protein 3 (RNA-binding motif protein 3) (RNPL)	RBM3 RNPL	0.04	0.95 ↓
P08559	Pyruvate dehydrogenase E1 component subunit alpha	PDHA1 PHE1A	0.04	0.97 ↓
Q9Y5J1	U3 small nucleolar RNA-associated protein 18 homolog	UTP18	0.04	0.96 ↓
P13010	X-ray repair cross-complementing protein 5	XRCC5	0.04	0.99 ↓
Q9H0S4	Probable ATP-dependent RNA helicase DDX47 (DEAD box protein 47)	DDX47	0.04	0.95 ↓
Q10713	Mitochondrial-processing peptidase subunit alpha (Alpha-MPP)	PMPCA INPP5E	0.04	0.98 ↓
P07910	Heterogeneous nuclear ribonucleoproteins C1/C2	HNRNPC	0.04	0.99 ↓
Q6UVY6	DBH-like monooxygenase protein 1	MOXD1	0.04	0.95 ↓
P35659	Protein DEK	DEK	0.04	0.98 ↓
Q9NZL4	Hsp70-binding protein 1 (HspBP1)	HSPBP1	0.04	1.02 ↑
P12814	Alpha-actinin-1	ACTN1	0.04	1.01 ↑
P62913	60S ribosomal protein L11	RPL11	0.04	1.01 ↑
Q32MZ4	Leucine-rich repeat flightless-interacting protein 1	LRRFIP1	0.04	1.04 ↑
P29317	Ephrin type-A receptor 2	EPHA2	0.04	1.02 ↑
Q99426	Tubulin-folding cofactor B	TBCB	0.04	1.06 ↑
Q16222	UDP-N-acetylhexosamine pyrophosphorylase (Antigen X) (AGX)	UAP1 SPAG2	0.04	1.02 ↑
P18669	Phosphoglycerate mutase 1	PGAM1	0.04	1.02 ↑
Q06323	Proteasome activator complex subunit 1	PSME1	0.04	1.03 ↑
P09936	Ubiquitin carboxyl-terminal hydrolase isozyme L1 (UCH-L1)	UCHL1	0.04	1.07 ↑
P09211	Glutathione S-transferase P	GSTP1	0.04	1.02 ↑
P04792	Heat shock protein beta-1 (HspB1)	HSPB1	0.04	1.01 ↑
Q9Y3F4	Serine-threonine kinase receptor-associated protein	STRAP	0.04	1.01 ↑
Q96FW1	Ubiquitin thioesterase OTUB1	OTUB1	0.04	1.07 ↑
O00154	Cytosolic acyl coenzyme A thioester hydrolase	ACOT7	0.03	1.02 ↑

Protein IDs	Protein Names	Gene IDs	*p*-value	Fold Change
Q04917	14-3-3 protein eta (Protein AS1)	YWHAH	0.03	1.02 ↑
P11413	Glucose-6-phosphate 1-dehydrogenase (G6PD)	G6PD	0.03	1.04 ↑
P23381	Tryptophan--tRNA ligase	WARS1	0.03	1.03 ↑
P11766	Alcohol dehydrogenase class-3	ADH5<br>ADHX	0.03	1.03 ↑
Q9NYL9	Tropomodulin-3 (Ubiquitous tropomodulin)	TMOD3	0.03	1.01 ↑
P68036	Ubiquitin-conjugating enzyme E2 L3	UBE2L3	0.03	1.03 ↑
P62266	40S ribosomal protein S23	RPS23	0.03	1.08 ↑
P67809	Y-box-binding protein 1 (YB-1)	YBX1	0.03	1.02 ↑
O95817	BAG family molecular chaperone regulator 3 (BAG-3)	BAG3<br>BIS	0.03	1.07 ↑
Q9NVA2	Septin-11	SEPTIN11<br>SEPT11	0.03	1.02 ↑
P31948	Stress-induced-phosphoprotein 1 (STI1)	STIP1	0.03	1.02 ↑
P34932	Heat shock 70 kDa protein 4 (HSP70RY)	HSPA4	0.03	1.02 ↑
Q02952	A-kinase anchor protein 12 (AKAP-12)	AKAP12	0.03	1.05 ↑
P49321	Nuclear autoantigenic sperm protein (NASP)	NASP	0.03	1.01 ↑
Q9H223	EH domain-containing protein 4	EHD4	0.03	1.02 ↑
P52788	Spermine synthase (SPMSY)	SMS	0.03	1.03 ↑
Q71U36	Tubulin alpha-1A chain (Alpha-tubulin 3)	TUBA1A	0.03	1.02 ↑
O75369	Filamin-B (FLN-B)	FLNB	0.02	1.01 ↑
P11142	Heat shock cognate 71 kDa protein	HSPA8<br>HSC70	0.02	1.01 ↑
P27695	DNA-(apurinic or apyrimidinic site) endonuclease	APEX1<br>APE	0.02	1.02 ↑
P49327	Fatty acid synthase	FASN/ FAS	0.02	1.02 ↑
P24666	Low molecular weight phosphotyrosine protein phosphatase (LMW-PTP)	ACP1	0.02	1.03 ↑
Q9UQ80	Proliferation-associated protein 2G4	PA2G4	0.02	1.02 ↑
P11908	Ribose-phosphate pyrophosphokinase 2	PRPS2	0.02	1.02 ↑
Q13501	Sequestosome-1 (EBI3-associated protein of 60 kDa) (EBIAP) (p60)	SQSTM1	0.02	1.05 ↑
P10768	S-formylglutathione hydrolase (FGH)	ESD	0.02	1.02 ↑
P60174	Triosephosphate isomerase (TIM)	TPI1/ TPI	0.02	1.03 ↑
Q9Y266	Nuclear migration protein nudC	NUDC	0.02	1.03 ↑
P59998	Actin-related protein 2/3 complex subunit 4	ARPC4	0.02	1.02 ↑
P35579	Myosin-9	MYH9	0.02	1.02 ↑
P46821	Microtubule-associated protein 1B (MAP-1B)	MAP1B	0.02	1.02 ↑
P08133	Annexin A6 (Annexin VI)	ANXA6	0.02	1.01 ↑

Protein IDs	Protein Names	Gene IDs	*p*-value	Fold Change
P27348	14-3-3 protein theta (14-3-3 protein T-cell)	YWHAQ	0.02	1.03 ↑
Q15181	Inorganic pyrophosphatase (EC 3.6.1.1) (Pyrophosphate phospho-hydrolase) (PPase)	PPA1	0.02	1.03 ↑
P62826	GTP-binding nuclear protein Ran	RAN	0.02	1.02 ↑
Q9Y2J8	Protein-arginine deiminase type-2	PADI2	0.02	1.02 ↑
Q99613	Eukaryotic translation initiation factor 3 subunit C (eIF3c)	EIF3C EIF3S8	0.02	1.00 ↑
P60983	Glia maturation factor beta (GMF-beta)	GMFB	0.02	1.03 ↑
P12268	Inosine-5'-monophosphate dehydrogenase 2 (IMP dehydrogenase 2) (IMPD 2)	IMPDH2 IMPD2	0.02	1.03 ↑
Q27J81	Inverted formin-2 (HBEBP2-binding protein C)	INF2	0.02	1.02 ↑
P61088	Ubiquitin-conjugating enzyme E2 N	UBE2N	0.02	1.03 ↑
P52907	F-actin-capping protein subunit alpha-1 (CapZ alpha-1)	CAPZA1	0.01	1.02 ↑
Q13442	28 kDa heat- and acid-stable phosphoprotein (PDGF-associated protein) (PAP)	PDAP1 HASPP28	0.01	1.05 ↑
Q15185	Prostaglandin E synthase 3	PTGES3	0.01	1.01 ↑
P35244	Replication protein A 14 kDa subunit (RP-A p14)	RPA3 RPA14	0.01	1.02 ↑
P50395	Rab GDP dissociation inhibitor beta (Rab GDI beta)	GDI2 RABGDIB	0.01	1.02 ↑
Q9BVG4	Protein PBDC1 (Polysaccharide biosynthesis domain-containing protein 1)	PBDC1 CXorf26	0.01	1.05 ↑
Q99733	Nucleosome assembly protein 1-like 4	NAP1L4	0.01	1.02 ↑
Q7Z2W4	Zinc finger CCCH-type antiviral protein 1	ZC3HAV1	0.01	1.02 ↑
Q96QK1	Vacuolar protein sorting-associated protein 35	VPS35	0.01	1.02 ↑
P06744	Glucose-6-phosphate isomerase (GPI)	GPI	0.01	1.02 ↑
Q9BZQ8	Protein Niban 1 (Protein FAM129A)	NIBAN1 FAM129A	0.01	1.03 ↑
P52209	6-phosphogluconate dehydrogenase, decarboxylating	PGD PGDH	0.01	1.02 ↑
Q9BRA2	Thioredoxin domain-containing protein 17	TXNDC17	0.01	1.04 ↑
P22061	Protein-L-isoaspartate(D-aspartate) O-methyltransferase (PIMT)	PCMT1	0.01	1.03 ↑
P23528	Cofilin-1	CFL1	0.01	1.02 ↑
P49591	Serine--tRNA ligase	SARS1	0.01	1.03 ↑
Q16658	Fascin	FSCN1	0.01	1.03 ↑
O00299	Chloride intracellular channel protein 1	CLIC1	0.009	1.03 ↑
P33316	Deoxyuridine 5'-triphosphate nucleotidohydrolase, mitochondrial (dUTPase)	DUT	0.009	1.01 ↑

Protein IDs	Protein Names	Gene IDs	*p*-value	Fold Change
P15311	Ezrin (Cytovillin) (Villin-2) (p81)	EZR VIL2	0.009	1.02 ↑
P30085	UMP-CMP kinase	CMPK1 CMK CMPK	0.009	1.03 ↑
O95218	Zinc finger Ran-binding domain-containing protein 2	ZRANB2	0.009	1.04 ↑
P31946	14-3-3 protein beta/alpha (Protein 1054) (Protein kinase C inhibitor protein 1) (KCIP-1)	YWHAB	0.008	1.02 ↑
Q8TAT6	Nuclear protein localization protein 4 homolog	NPLOC4	0.008	1.03 ↑
P30086	Phosphatidylethanolamine-binding protein 1 (PEBP-1)	PEBP1/ PBP/ PEBP	0.008	1.04 ↑
P30041	Peroxiredoxin-6	PRDX6	0.008	1.02 ↑
P46940	Ras GTPase-activating-like protein IQGAP1 (p195)	IQGAP1 KIAA0051	0.008	1.01 ↑
P52565	Rho GDP-dissociation inhibitor 1 (Rho GDI 1)	ARHGDIA	0.007	1.04 ↑
O60664	Perilipin-3	PLIN3	0.007	1.04 ↑
P41250	Glycine--tRNA ligase	GARS1 GARS	0.007	1.03 ↑
P11586	C-1-tetrahydrofolate synthase, Methylenetetrahydrofolate dehydrogenase	MTHFD1 MTHFC MTHFD	0.007	1.02 ↑
P31150	Rab GDP dissociation inhibitor alpha (Rab GDI alpha)	GDI1 GDIL	0.007	1.04 ↑
P50990	T-complex protein 1 subunit theta (TCP-1-theta)	CCT8 CCTQ	0.007	1.01 ↑
P37802	Transgelin-2 (Epididymis tissue protein Li 7e)	TAGLN2	0.007	1.02 ↑
Q14019	Coactosin-like protein	COTL1	0.007	1.04 ↑
Q15102	Platelet-activating factor acetylhydrolase IB subunit alpha1	PAFAH1B3	0.006	1.02 ↑
P06733	Alpha-enolase	ENO1	0.006	1.03 ↑
P21291	Cysteine and glycine-rich protein 1	CSRP1 CSRP	0.006	1.05 ↑
P04083	Annexin A1 (Annexin I)	ANXA1	0.006	1.02 ↑
P39748	Flap endonuclease 1 (FEN-1)	FEN1 RAD2	0.005	1.03 ↑
P84077	ADP-ribosylation factor 1	ARF1	0.005	1.02 ↑
P67936	Tropomyosin alpha-4 chain (TM30p1)	TPM4	0.005	1.02 ↑
P22234	Multifunctional protein ADE2	PAICS ADE2	0.005	1.02 ↑
P20042	Eukaryotic translation initiation factor 2 subunit 2	EIF2S2	0.004	1.02 ↑

Protein IDs	Protein Names	Gene IDs	*p*-value	Fold Change
Q9BUF5	Tubulin beta-6 chain	TUBB6	0.004	1.03 ↑
Q14192	Four and a half LIM domains protein 2 (FHL-2)	FHL2	0.003	1.03 ↑
O95373	Importin-7 (Imp7)	IPO7	0.003	1.01 ↑
O43707	Alpha-actinin-4 (Non-muscle alpha-actinin 4)	ACTN4	0.003	1.01 ↑
O43175	D-3-phosphoglycerate dehydrogenase (3-PGDH)	PHGDH PGDH3	0.003	1.03 ↑
P27694	Replication protein A 70 kDa DNA-binding subunit	RPA1 RPA70	0.003	1.03 ↑
P14618	Pyruvate kinase PKM	PKM/ OIP3	0.003	1.02 ↑
P16949	Stathmin	STMN1	0.002	1.02 ↑
Q99497	Parkinson disease protein 7	PARK7	0.002	1.04 ↑
P15559	NAD(P)H dehydrogenase [quinone] 1	NQO1	0.001	1.03 ↑
Q92945	Far upstream element-binding protein 2 (FUSE-binding protein 2)	KHSRP FUBP2	0.001	1.01 ↑
P05387	60S acidic ribosomal protein P2	RPLP2	0.001	1.01 ↑
P05388	60S acidic ribosomal protein P0	RPLP0	0.0010	1.01 ↑
P49773	Histidine triad nucleotide-binding protein 1	HINT1	0.0009	1.03 ↑
P50502	Hsc70-interacting protein (Hip)	ST13 FAM10A1	0.0009	1.02 ↑
P68366	Tubulin alpha-4A chain (Alpha-tubulin 1)	TUBA4A	0.0007	1.03 ↑
P21333	Filamin-A (FLN-A)	FLNA/ FLN	0.0007	1.02 ↑
P07195	L-lactate dehydrogenase B chain (LDH-B)	LDHB	0.0005	1.03 ↑
Q02790	Peptidyl-prolyl cis-trans isomerase FKBP4 (PPIase FKBP4)	FKBP4 FKBP52	0.0005	1.02 ↑
P23526	Adenosylhomocysteinase (AdoHcyase)	AHCY	0.0004	1.03 ↑
P08758	Annexin A5 (Annexin V)	ANXA5	0.0003	1.02 ↑
P32119	Peroxiredoxin-2	PRDX2	0.0003	1.03 ↑
P40925	Malate dehydrogenase	MDH1	0.0002	1.02 ↑
P63104	14-3-3 protein zeta/delta (Protein kinase C inhibitor protein 1) (KCIP-1)	YWHAZ	0.0002	1.03 ↑
Q9NR12	PDZ and LIM domain protein 7	PDLIM7	0.0001	1.03 ↑
P22102	Trifunctional purine biosynthetic protein adenosine-3	GART	0.00009	1.03 ↑

Table S3. List of altered proteins in U118 cells versus Control, after treatment with 0.5 µM ST1926 for 2 hours. A total of 96 proteins with significant changes (45 downregulated (↓) and 51 upregulated (↑)) were identified in U118 cells with $p < 0.05$.

Protein IDs	Protein Names	Gene IDs	*p*-value	Fold Change
Q96TA1	Protein Niban 2(Protein FAM129B)	NIBAN2<br>FAM129B	0.00004	0.97 ↓
P52294	Importin subunit alpha-5	KPNA1	0.0006	0.91 ↓
P26641	Elongation factor 1-gamma	EEF1G<br>EF1G	0.001	0.99 ↓
P11216	Glycogen phosphorylase	PYGB	0.002	0.99 ↓
Q99733	Nucleosome assembly protein 1-like 4	NAP1L4	0.003	0.98 ↓
P11388	DNA topoisomerase 2-alpha	TOP2A<br>TOP2	0.003	0.96 ↓
Q15417	Calponin-3	CNN3	0.004	0.97 ↓
P46109	Crk-like protein	CRKL	0.007	0.99 ↓
P31939	Bifunctional purine biosynthesis protein ATIC (AICAR transformylase/inosine monophosphate cyclohydrolase)	ATIC<br>PURH	0.01	0.99 ↓
Q14192	Four and a half LIM domains protein 2 (FHL-2)	FHL2	0.01	0.99 ↓
P17301	Integrin alpha-2 (CD49 antigen-like family member B)	ITGA2<br>CD49B	0.01	0.97 ↓
P62851	40S ribosomal protein S25	RPS25	0.01	0.98 ↓
P08708	40S ribosomal protein S17	RPS17	0.01	0.98 ↓
P61289	Proteasome activator complex subunit 3 (11S regulator complex subunit gamma)	PSME3	0.02	0.99 ↓
P55060	Exportin-2 (Exp2)	XPO2	0.02	0.99 ↓
P53004	Biliverdin reductase A (BVR A)	BLVRA<br>BLVR	0.02	0.98 ↓
P31150	Rab GDP dissociation inhibitor alpha (Rab GDI alpha)	GDI1<br>GDIL	0.02	0.98 ↓
Q14204	Cytoplasmic dynein 1 heavy chain 1	DYNC1H1	0.02	0.99 ↓
Q8NBT2	Kinetochore protein Spc24	SPC24	0.02	0.96 ↓
Q96C19	EF-hand domain-containing protein D2	EFHD2	0.02	0.99 ↓
Q9H0S4	Probable ATP-dependent RNA helicase DDX47 (DEAD box protein 47)	DDX47	0.03	0.99 ↓
P62081	40S ribosomal protein S7	RPS7	0.03	0.97 ↓
Q14157	Ubiquitin-associated protein 2-like	UBAP2L	0.03	0.91 ↓
Q96HC4	PDZ and LIM domain protein 5	PDLIM5	0.03	0.96 ↓
P14618	Pyruvate kinase PKM	PKM/ OIP3	0.03	0.99 ↓

Protein IDs	Protein Names	Gene IDs	*p*-value	Fold Change
P24534	Elongation factor 1-beta (EF-1-beta)	EEF1B2 EEF1B	0.03	0.99 ↓
P02751	Fibronectin (FN)	FN1 FN	0.04	0.93 ↓
Q9BUF5	Tubulin beta-6 chain	TUBB6	0.04	0.97 ↓
P17612	cAMP-dependent protein kinase catalytic subunit alpha (PKA C-alpha)	PRKACA PKACA	0.04	0.96 ↓
Q9BVG4	Protein PBDC1 (Polysaccharide biosynthesis domain-containing protein 1)	PBDC1 CXorf26	0.04	0.97 ↓
P27708	CAD protein	CAD	0.04	0.99 ↓
Q14008	Cytoskeleton-associated protein 5	CKAP5	0.04	0.98 ↓
P16403	Histone H1.2	H1-2 HIST1H1C	0.04	0.98 ↓
Q01813	ATP-dependent 6-phosphofructokinase	PFKP PFKF	0.04	0.99 ↓
Q9NTK5	Obg-like ATPase 1	OLA1	0.04	0.99 ↓
P50991	T-complex protein 1 subunit delta (TCP-1-delta)	CCT4 CCTD	0.04	0.99 ↓
P52888	Thimet oligopeptidase	THOP1	0.04	0.98 ↓
Q96T76	MMS19 nucleotide excision repair protein homolog (hMMS19)	MMS19 MMS19L	0.04	0.98 ↓
P00492	Hypoxanthine-guanine phosphoribosyltransferase (HGPRT)	HPRT1 HPRT	0.04	0.97 ↓
P48643	T-complex protein 1 subunit epsilon (TCP-1-epsilon)	CCT5 CCTE	0.040	0.99 ↓
P46940	Ras GTPase-activating-like protein IQGAP1 (p195)	IQGAP1 KIAA0051	0.040	0.99 ↓
P68371	Tubulin beta-4B chain (Tubulin beta-2 chain)	TUBB4B TUBB2C	0.040	0.97 ↓
P35637	RNA-binding protein FUS	FUS TLS	0.040	0.99 ↓
P22234	Multifunctional protein ADE2	PAICS ADE2	0.04	0.98 ↓
Q6UVK1	Chondroitin sulfate proteoglycan 4	CSPG4	0.04	0.99 ↓
Q9Y5L4	Mitochondrial import inner membrane translocase subunit Tim13	TIMM13	0.04	1.01 ↑
Q15393	Splicing factor 3B subunit 3	SF3B3	0.04	1.01 ↑
Q9NZ01	Very-long-chain enoyl-CoA reductase	TECR	0.04	1.02 ↑
Q14103	Heterogeneous nuclear ribonucleoprotein D0 (hnRNP D0)	HNRNPD AUF1	0.04	1.01 ↑
P35579	Myosin-9	MYH9	0.04	1.01 ↑
Q14566	DNA replication licensing factor MCM6	MCM6	0.04	1.02 ↑
P10809	60 kDa heat shock protein (Chaperonin 60)	HSPD1 HSP60	0.04	1.01 ↑

Protein IDs	Protein Names	Gene IDs	*p*-value	Fold Change
P17931	Galectin-3 (Gal-3)	LGALS3	0.04	1.01 ↑
P67812	Signal peptidase complex catalytic subunit SEC11A	SEC11A	0.04	1.02 ↑
P38117	Electron transfer flavoprotein subunit beta (Beta-ETF)	ETFB FP585	0.04	1.02 ↑
Q8WWM7	Ataxin-2-like protein	ATXN2L	0.04	1.03 ↑
Q12888	TP53-binding protein 1 (p53BP1)	TP53BP1	0.04	1.02 ↑
P02786	Transferrin receptor protein 1 (TfR1)	TFRC	0.03	1.01 ↑
P09972	Fructose-bisphosphate aldolase C	ALDOC	0.03	1.01 ↑
P14625	Endoplasmin	HSP90B1	0.03	1.01 ↑
P10620	Microsomal glutathione S-transferase 1 (Microsomal GST-1)	MGST1 GST12	0.03	1.03 ↑
O75533	Splicing factor 3B subunit 1	SF3B1	0.03	1.01 ↑
Q8NBJ5	Procollagen galactosyltransferase 1	COLGALT 1	0.03	1.02 ↑
P51659	Peroxisomal multifunctional enzyme type 2 (MFE-2)	HSD17B4	0.03	1.02 ↑
O60443	Gasdermin-E	GSDME	0.03	1.01 ↑
P52597	Heterogeneous nuclear ribonucleoprotein F (hnRNP F)	HNRNPF HNRPF	0.03	1.01 ↑
Q92878	DNA repair protein RAD50 (hRAD50)	RAD50	0.03	1.01 ↑
Q96N66	Lysophospholipid acyltransferase 7 (LPLAT 7)	MBOAT7	0.03	1.01 ↑
P35232	Prohibitin	PHB	0.03	1.01 ↑
Q99798	Aconitate hydratase (Aconitase)	ACO2	0.02	1.01 ↑
P30519	Heme oxygenase 2 (HO-2)	HMOX2 HO2	0.02	1.02 ↑
P34897	Serine hydroxymethyltransferase (SHMT)	SHMT2	0.02	1.01 ↑
P11021	Endoplasmic reticulum chaperone BiP	HSPA5	0.02	1.02 ↑
P14866	Heterogeneous nuclear ribonucleoprotein L (hnRNP L)	HNRNPL HNRPL	0.02	1.01 ↑
P25788	Proteasome subunit alpha type-3	PSMA3	0.02	1.02 ↑
O75396	Vesicle-trafficking protein SEC22b	SEC22B	0.02	1.03 ↑
P30048	Thioredoxin-dependent peroxide reductase	PRDX3	0.02	1.01 ↑
O95817	BAG family molecular chaperone regulator 3 (BAG-3)	BAG3 BIS	0.02	1.01 ↑
Q15907	Ras-related protein Rab-11B	RAB11B	0.01	1.01 ↑
Q15424	Scaffold attachment factor B1 (SAF-B)	SAFB HAP	0.01	1.02 ↑
O15258	Protein RER1	RER1	0.01	1.02 ↑

**Protein IDs**	**Protein Names**	**Gene IDs**	***p*-value**	**Fold Change**
P05413	Fatty acid-binding protein 3	FABP3	0.01	1.02 ↑
O00264	Membrane-associated progesterone receptor component 1 (mPR)	PGRMC1	0.009	1.03 ↑
P23284	Peptidyl-prolyl cis-trans isomerase B (PPIase B)	PPIB	0.008	1.01 ↑
P37837	Transaldolase	TALDO1	0.008	1.02 ↑
P61421	V-type proton ATPase subunit d 1 (V-ATPase subunit d 1)	ATP6V0D1 ATP6D	0.007	1.01 ↑
Q15459	Splicing factor 3A subunit 1 (SF3a120)	SF3A1	0.006	1.02 ↑
P50416	Carnitine O-palmitoyltransferase 1 (CPT1-L)	CPT1A	0.006	1.01 ↑
P02545	Prelamin-A/C	LMNA	0.006	1.01 ↑
P38646	Stress-70 protein (Heat shock 70 kDa protein 9)	HSPA9	0.005	1.01 ↑
Q92841	Probable ATP-dependent RNA helicase DDX17 (DEAD box protein 17)	DDX17	0.005	1.01 ↑
P40926	Malate dehydrogenase	MDH2	0.005	1.01 ↑
P51148	Ras-related protein Rab-5C (RAB5L)	RAB5C	0.004	1.02 ↑
Q13011	Delta(3,5)-Delta(2,4)-dienoyl-CoA isomeras, mitochondrial (EC 5.3.3.-)	ECH1	0.004	1.02 ↑
O94979	Protein transport protein Sec31A	SEC31A	0.001	1.01 ↑
P04216	Thy-1 membrane glycoprotein	THY1	0.0006	1.03 ↑

Table S4. List of altered proteins in U118 cells versus Control, after treatment with 0.5 µM ST1926 for 24 hours. A total of 71 proteins with significant changes (35 downregulated (↓) and 36 upregulated (↑)) were identified in U118 cells with $p < 0.05$.

Protein IDs	Protein Names	Gene IDs	*p*-value	Fold Change
P11388	DNA topoisomerase 2-alpha	TOP2A TOP2	0.0001	0.95 ↓
P52294	Importin subunit alpha-5	KPNA1	0.0006	0.91 ↓
Q5SSJ5	Heterochromatin protein 1-binding protein 3	HP1BP3	0.001	0.98 ↓
P35221	Catenin alpha-1 (Alpha E-catenin)	CTNNA1	0.004	0.99 ↓
P04899	Guanine nucleotide-binding protein G(i) subunit alpha-2	GNAI2 GNAI2B	0.006	0.98 ↓
P62979	Ubiquitin-40S ribosomal protein S27a	RPS27A	0.007	0.98 ↓
Q96TA1	Protein Niban 2 (Protein FAM129B)	NIBAN2 FAM129B	0.007	0.98 ↓
Q01082	Spectrin beta chain	SPTBN1	0.008	0.99 ↓
Q53GQ0	Very-long-chain 3-oxoacyl-CoA reductase	HSD17B12	0.01	0.98 ↓
P12109	Collagen alpha-1(VI) chain	COL6A1	0.01	0.99 ↓
P12111	Collagen alpha-3(VI) chain	COL6A3	0.01	0.97 ↓
Q13148	TAR DNA-binding protein 43 (TDP-43)	TARDBP TDP43	0.01	0.99 ↓
P40763	Signal transducer and activator of transcription 3	STAT3 APRF	0.01	0.98 ↓
O75534	Cold shock domain-containing protein E1	CSDE1	0.02	0.99 ↓
P04181	Ornithine aminotransferase	OAT	0.02	0.98 ↓
Q09666	Neuroblast differentiation-associated protein AHNAK (Desmoyokin)	AHNAK PM227	0.02	0.99 ↓
Q01813	ATP-dependent 6-phosphofructokinase	PFKP	0.03	0.99 ↓
Q99733	Nucleosome assembly protein 1-like 4	NAP1L4	0.03	0.99 ↓
Q9Y5L0	Transportin-3	TNPO3	0.03	0.98 ↓
P62273	40S ribosomal protein S29	RPS29	0.03	0.98 ↓
O14980	Exportin-1 (Exp1)	XPO1	0.03	0.99 ↓
Q9BTT0	Acidic leucine-rich nuclear phosphoprotein 32 family member E (LANP-like protein)	ANP32E	0.03	0.99 ↓
P47756	F-actin-capping protein subunit beta (CapZ beta)	CAPZB	0.03	0.99 ↓
P49773	Histidine triad nucleotide-binding protein 1	HINT1	0.04	0.96 ↓
Q5T4S7	E3 ubiquitin-protein ligase UBR4	UBR4	0.04	0.98 ↓
P49720	Proteasome subunit beta type-3	PSMB3	0.04	0.99 ↓
Q9UH99	SUN domain-containing protein 2	SUN2	0.04	0.96 ↓
P20073	Annexin A7	ANXA7 ANX7	0.04	0.99 ↓

Protein IDs	Protein Names	Gene IDs	*p*-value	Fold Change
Q96P70	Importin-9 (Imp9)	IPO9	0.04	0.98 ↓
O76094	Signal recognition particle subunit SRP72	SRP72	0.04	0.98 ↓
Q6IBS0	Twinfilin-2	TWF2	0.04	0.97 ↓
P31939	Bifunctional purine biosynthesis protein ATIC (AICAR transformylase/inosine monophosphate cyclohydrolase)	ATIC PURH	0.04	0.99 ↓
O60568	Multifunctional procollagen lysine hydroxylase and glycosyltransferase LH3	PLOD3	0.04	0.97 ↓
P42224	Signal transducer and activator of transcription 1-alpha/beta	STAT1	0.04	0.99 ↓
P12956	X-ray repair cross-complementing protein 6	XRCC6	0.04	0.98 ↓
P26641	Elongation factor 1-gamma (EF-1-gamma)	EEF1G EF1G	0.04	1.01 ↑
P13473	Lysosome-associated membrane glycoprotein 2 (LAMP-2)	LAMP2	0.04	1.01 ↑
P11021	Endoplasmic reticulum chaperone BiP	HSPA5	0.04	1.02 ↑
P55084	Trifunctional enzyme subunit beta	HADHB	0.04	1.01 ↑
P54819	Adenylate kinase 2 (AK 2)	AK2 ADK2	0.04	1.01 ↑
P17931	Galectin-3 (Gal-3)	LGALS3	0.04	1.02 ↑
Q9BYG3	MKI67 FHA domain-interacting nucleolar phosphoprotein	NIFK MKI67IP	0.04	1.04 ↑
P14625	Endoplasmin	HSP90B1	0.04	1.01 ↑
Q9P2J5	Leucine--tRNA ligase	LARS1	0.04	1.01 ↑
P49411	Elongation factor Tu (EF-Tu)	TUFM	0.04	1.00 ↑
Q8NBJ5	Procollagen galactosyltransferase 1	COLGALT 1	0.03	1.01 ↑
O43237	Cytoplasmic dynein 1 light intermediate chain 2	DYNC1LI2	0.03	1.01 ↑
Q8N6T3	ADP-ribosylation factor GTPase-activating protein 1 (ARF GAP 1)	ARFGAP1 ARF1GAP	0.03	1.02 ↑
P68366	Tubulin alpha-4A chain (Alpha-tubulin 1)	TUBA4A	0.03	1.01 ↑
P51148	Ras-related protein Rab-5C (RAB5L)	RAB5C	0.03	1.02 ↑
P41252	Isoleucine--tRNA ligase	IARS1	0.03	1.01 ↑
P30519	Heme oxygenase 2 (HO-2)	HMOX2 HO2	0.03	1.02 ↑
Q14566	DNA replication licensing factor MCM6	MCM6	0.03	1.02 ↑
P23284	Peptidyl-prolyl cis-trans isomerase B (PPIase B)	PPIB	0.03	1.02 ↑
Q15459	Splicing factor 3A subunit 1 (SF3a120)	SF3A1	0.03	1.01 ↑
P08195	4F2 cell-surface antigen heavy chain (4F2hc)	SLC3A2	0.02	1.02 ↑
P38646	Stress-70 protein (Heat shock 70 kDa protein 9)	HSPA9	0.02	1.01 ↑
P49756	RNA-binding protein 25	RBM25	0.02	1.03 ↑

Protein IDs	Protein Names	Gene IDs	*p*-value	Fold Change
Q13501	Sequestosome-1 (EBI3-associated protein of 60 kDa) (EBIAP) (p60)	SQSTM1	0.02	1.03 ↑
Q9P0L0	Vesicle-associated membrane protein-associated protein A (VAMP-A)	VAPA VAP33	0.02	1.01 ↑
Q9P258	Protein RCC2 (RCC1-like protein TD-60)	RCC2	0.02	1.01 ↑
P52597	Heterogeneous nuclear ribonucleoprotein F (hnRNP F)	HNRNPF HNRPF	0.02	1.02 ↑
P34897	Serine hydroxymethyltransferase (SHMT)	SHMT2	0.02	1.01 ↑
Q96T88	E3 ubiquitin-protein ligase UHRF1	UHRF1	0.02	1.02 ↑
Q13838	Spliceosome RNA helicase DDX39B	DDX39B	0.01	1.00 ↑
P23526	Adenosylhomocysteinase (AdoHcyase)	AHCY	0.01	1.01 ↑
P02545	Prelamin-A/C	LMNA	0.01	1.01 ↑
P04216	Thy-1 membrane glycoprotein	THY1	0.006	1.02 ↑
P05413	Fatty acid-binding protein	FABP3	0.004	1.03 ↑
P37837	Transaldolase	TALDO1	0.004	1.02 ↑
P35232	Prohibitin	PHB	0.002	1.01 ↑

Table S5. List of altered proteins in U118 cells versus Control, after treatment with 0.5 µM ST1926 for 48 hours. A total of 136 proteins with significant changes (53 downregulated (↓) and 83 upregulated (↑)) were identified in U118 cells with $p < 0.05$.

**Protein IDs**	**Protein Names**	**Gene IDs**	***p*-value**	**Fold Change**
P04181	Ornithine aminotransferase, mitochondrial	OAT	0.001	0.98 ↓
P40763	Signal transducer and activator of transcription 3	STAT3	0.002	0.98 ↓
P12109	Collagen alpha-1(VI) chain	CO6A1	0.003	0.98 ↓
Q09666	Neuroblast differentiation-associated protein AHNAK	AHNK	0.004	0.99 ↓
P06753	Tropomyosin alpha-3 chain	TPM3	0.004	0.98 ↓
Q6UVK1	Chondroitin sulfate proteoglycan 4	CSPG4	0.004	0.98 ↓
P40227	T-complex protein 1 subunit zeta (TCP-1-zeta)	TCPZ	0.006	0.98 ↓
Q7Z4H8	Protein O-glucosyltransferase 3	PLGT3	0.006	0.99 ↓
Q13185	Chromobox protein homolog 3	CBX3	0.007	0.97 ↓
Q8NBT2	Kinetochore protein Spc24	SPC24	0.008	0.95 ↓
Q9P2E9	Ribosome-binding protein 1	RRBP1	0.009	0.98 ↓
P12111	Collagen alpha-3 (VI) chain	CO6A3	0.009	0.95 ↓
O00571	ATP-dependent RNA helicase DDX3X	DDX3X	0.01	0.98 ↓
P15144	Aminopeptidase N (AP-N)	AMPN	0.01	0.99 ↓
Q96QK1	Vacuolar protein sorting-associated protein 35 (hVPS35)	VPS35	0.01	0.99 ↓
Q6YHK3	CD109 antigen	CD109	0.01	0.96 ↓
Q14980	Nuclear mitotic apparatus protein 1	NUMA1	0.01	0.99 ↓
Q5SSJ5	Heterochromatin protein 1-binding protein 3	HP1B3	0.02	0.98 ↓
Q99685	Monoglyceride lipase (MGL)	MGLL	0.02	0.99 ↓
Q9BUF5	Tubulin beta-6 chain (Tubulin beta class V)	TBB6	0.02	0.97 ↓
P53618	Coatomer subunit beta	COPB	0.02	0.98 ↓
P26358	DNA (cytosine-5)-methyltransferase 1 (Dnmt1)	DNMT1	0.02	0.96 ↓
Q7L1Q6	eIF5-mimic protein 2	BZW1	0.02	0.98 ↓
P48643	T-complex protein 1 subunit epsilon (TCP-1-epsilon)	TCPE	0.02	0.99 ↓
P13010	X-ray repair cross-complementing protein 5	XRCC5	0.02	0.99 ↓
O00186	Syntaxin-binding protein 3	STXB3	0.02	0.98 ↓
P04899	Guanine nucleotide-binding protein G(i) subunit alpha-2	GNAI2	0.02	0.98 ↓
Q07666	KH domain-containing, RNA-binding, signal transduction-associated protein 1 (GAP-associated tyrosine phosphoprotein p62)	KHDR1	0.02	0.95 ↓
Q96TA1	Protein Niban 2	NIBL1	0.02	0.97 ↓

Protein IDs	Protein Names	Gene IDs	*p*-value	Fold Change
P12956	X-ray repair cross-complementing protein 6	XRCC6	0.02	0.98 ↓
Q9Y3A5	Ribosome maturation protein SBDS	SBDS	0.02	0.99 ↓
P80303	Nucleobindin-2	NUCB2	0.02	0.96 ↓
P42224	Signal transducer and activator of transcription 1-alpha/beta	STAT1	0.02	0.98 ↓
O75534	Cold shock domain-containing protein E1	CSDE1	0.03	0.99 ↓
Q9BW60	Elongation of very long chain fatty acids protein 1	ELOV1	0.03	0.98 ↓
O75506	Heat shock factor-binding protein 1	HSBP1	0.03	0.95 ↓
P11388	DNA topoisomerase 2-alpha	TOP2A	0.03	0.92 ↓
Q01082	Spectrin beta chain, non-erythrocytic 1	SPTB2	0.03	0.99 ↓
O14579	Coatomer subunit epsilon	COPE	0.03	0.98 ↓
Q9UHD8	Septin-9	SEPT9.	0.03	0.98 ↓
O60568	Multifunctional procollagen lysine hydroxylase and glycosyltransferase LH3	PLOD3	0.03	0.97 ↓
P52292	Importin subunit alpha-1	IMA1	0.04	0.87 ↓
P13639	Elongation factor 2 (EF-2)	EF2	0.04	0.99 ↓
P02751	Fibronectin (FN)	FINC	0.04	0.91 ↓
P52209	6-phosphogluconate dehydrogenase, decarboxylating	6PGD	0.04	0.99 ↓
Q9UH99	SUN domain-containing protein 2	SUN2	0.04	0.96 ↓
Q9Y5K5	Ubiquitin carboxyl-terminal hydrolase isozyme L5 (UCH-L5)	UCHL5	0.04	0.99 ↓
P07711	Procathepsin L	CATL1	0.04	0.95 ↓
O94925	Glutaminase kidney isoform, mitochondrial (GLS)	GLSK	0.04	0.99 ↓
Q9NR30	Nucleolar RNA helicase 2	DDX21	0.04	0.98 ↓
P24752	Acetyl-CoA acetyltransferase, mitochondrial	THIL	0.04	0.99 ↓
P17844	Probable ATP-dependent RNA helicase DDX5	DDX5	0.04	0.98 ↓
P47756	F-actin-capping protein subunit beta (CapZ beta)	CAPZB	0.04	0.99 ↓
Q92841	Probable ATP-dependent RNA helicase DDX17	DDX17	0.04	1.01 ↑
P09960	Leukotriene A-4 hydrolase (LTA-4 hydrolase)	LKHA4	0.04	1.01 ↑
P39748	Flap endonuclease 1 (FEN-1)	FEN1	0.04	1.02 ↑
O43615	Mitochondrial import inner membrane translocase subunit TIM44	TIM44	0.04	1.07 ↑
Q96T88	E3 ubiquitin-protein ligase UHRF1	UHRF1	0.04	1.01 ↑
P40938	Replication factor C subunit 3	RFC3	0.04	1.01 ↑
Q92878	DNA repair protein RAD50 (hRAD50)	RAD50	0.04	1.01 ↑

Protein IDs	Protein Names	Gene IDs	*p*-value	Fold Change
Q13838	Spliceosome RNA helicase DDX39B	DX39B	0.04	1.01 ↑
P38117	Electron transfer flavoprotein subunit beta (Beta-ETF)	ETFB	0.04	1.02 ↑
P53992	Protein transport protein Sec24C	SC24C	0.04	1.01 ↑
Q9NZB2	Constitutive coactivator of PPAR-gamma-like protein 1	F120A	0.04	1.02 ↑
P11940	Polyadenylate-binding protein 1 (PABP-1)	PABP1	0.04	1.01 ↑
Q12888	TP53-binding protein 1 (53BP1) (p53-binding protein 1)	TP53B	0.04	1.01 ↑
Q14103	Heterogeneous nuclear ribonucleoprotein D0 (hnRNP D0)	HNRPD	0.04	1.01 ↑
P30086	Phosphatidylethanolamine-binding protein 1 (PEBP-1)	PEBP1	0.03	1.01 ↑
O75369	Filamin-B (FLN-B)	FLNB	0.03	1.01 ↑
P31153	S-adenosylmethionine synthase isoform type-2 (AdoMet synthase 2)	METK2	0.03	1.02 ↑
P45880	Voltage-dependent anion-selective channel protein 2 (VDAC-2)	VDAC2	0.03	1.01 ↑
P50416	Carnitine O-palmitoyltransferase 1, liver isoform (CPT1-L)	CPT1A	0.03	1.01 ↑
Q9Y3E5	Peptidyl-tRNA hydrolase 2, mitochondrial (PTH 2)	PTH2	0.03	1.02 ↑
P30041	Peroxiredoxin-6	PRDX6	0.03	1.02 ↑
P51659	Peroxisomal multifunctional enzyme type 2 (MFE-2)	DHB4	0.03	1.02 ↑
P11413	Glucose-6-phosphate 1-dehydrogenase (G6PD)	G6PD	0.03	1.02 ↑
P18859	ATP synthase-coupling factor 6, mitochondrial (ATPase subunit F6)	ATP5J	0.03	1.02 ↑
P22307	Sterol carrier protein 2 (SCP-2)	NLTP	0.03	1.02 ↑
O43175	D-3-phosphoglycerate dehydrogenase (3-PGDH)	SERA	0.03	1.02 ↑
P21796	Voltage-dependent anion-selective channel protein 1 (VDAC-1)	VDAC1	0.03	1.01 ↑
Q93052	Lipoma-preferred partner	LPP	0.03	1.02 ↑
Q9BV86	N-terminal Xaa-Pro-Lys N-methyltransferase 1	NTM1A	0.03	1.02 ↑
Q92945	Far upstream element-binding protein 2	FUBP2	0.03	1.02 ↑
O43592	Exportin-T	XPOT	0.03	1.02 ↑
P36776	Lon protease homolog, mitochondrial	LONM	0.03	1.01 ↑
P33992	DNA replication licensing factor MCM5	MCM5	0.03	1.02 ↑
P23284	Peptidyl-prolyl cis-trans isomerase B (PPIase B)	PPIB	0.03	1.02 ↑
Q9P258	Protein RCC2 (RCC1-like protein TD-60)	RCC2	0.02	1.01 ↑

Protein IDs	Protein Names	Gene IDs	*p*-value	Fold Change
P14314	Glucosidase 2 subunit beta (80K-H protein)	GLU2B	0.02	1.01 ↑
P60174	Triosephosphate isomerase (TIM)	TPIS	0.02	1.01 ↑
P54577	Tyrosine--tRNA ligase, cytoplasmic	SYYC	0.02	1.01 ↑
P16152	Carbonyl reductase [NADPH] 1	CBR1	0.02	1.01 ↑
Q16543	Hsp90 co-chaperone Cdc37 (Hsp90 chaperone protein kinase-targeting subunit) (p50Cdc37)	CDC37	0.02	1.01 ↑
P55084	Trifunctional enzyme subunit beta, mitochondrial (TP-beta)	ECHB	0.02	1.01 ↑
Q9UQ80	Proliferation-associated protein 2G4 (Cell cycle protein p38-2G4 homolog) (hG4-1)	PA2G4	0.02	1.01 ↑
P21980	Protein-glutamine gamma-glutamyltransferase 2	TGM2	0.02	1.04 ↑
P04083	Annexin A1 (Annexin I)	ANXA1	0.02	1.01 ↑
O95831	Apoptosis-inducing factor 1, mitochondrial	AIFM1	0.02	1.02 ↑
Q14566	DNA replication licensing factor MCM6	MCM6	0.02	1.02 ↑
Q05682	Caldesmon (CDM)	CALD1	0.02	1.03 ↑
O75340	Programmed cell death protein 6 (Apoptosis-linked gene 2 protein homolog) (ALG-2)	PDCD6	0.02	1.02 ↑
P29401	Transketolase (TK)	TKT	0.02	1.01 ↑
P62633	CCHC-type zinc finger nucleic acid binding protein	CNBP	0.01	1.02 ↑
Q08211	ATP-dependent RNA helicase A	DHX9	0.01	1.01 ↑
P30043	Flavin reductase (NADPH) (FR)	BLVRB	0.01	1.04 ↑
P32119	Peroxiredoxin-2	PRDX2	0.01	1.02 ↑
P05387	Large ribosomal subunit protein P2	RLA2	0.01	1.01 ↑
P07602	Prosaposin (Proactivator polypeptide)	SAP	0.01	1.01 ↑
Q16527	Cysteine and glycine-rich protein 2 (Cysteine-rich protein 2) (CRP2)	CSRP2	0.01	1.06 ↑
P46459	Vesicle-fusing ATPase	NSF	0.01	1.01 ↑
P11021	Endoplasmic reticulum chaperone BiP	BIP	0.01	1.02 ↑
P29692	Elongation factor 1-delta (EF-1-delta)	EF1D	0.009	1.01 ↑
O94979	Protein transport protein Sec31A (ABP125)	SC31A	0.008	1.02 ↑
P54819	Adenylate kinase 2, mitochondrial (AK 2)	KAD2	0.008	1.02 ↑
Q13011	Delta(3,5)-Delta(2,4)-dienoyl-CoA isomerase, mitochondrial	ECH1	0.007	1.02 ↑
Q14192	Four and a half LIM domains protein 2 (FHL-2)	FHL2	0.007	1.03 ↑
P68366	Tubulin alpha-4A chain	TBA4A	0.006	1.02 ↑
P09972	Fructose-bisphosphate aldolase C (Brain-type aldolase)	ALDOC	0.006	1.02 ↑

**Protein IDs**	**Protein Names**	**Gene IDs**	***p*-value**	**Fold Change**
Q9BRA2	Thioredoxin domain-containing protein 17 (14 kDa thioredoxin-related protein) (TRP14)	TXD17	0.006	1.03 ↑
O75396	Vesicle-trafficking protein SEC22b (ER-Golgi SNARE of 24 kDa) (ERS-24)	SC22B	0.005	1.03 ↑
O75533	Splicing factor 3B subunit 1 (Pre-mRNA-splicing factor SF3b 155 kDa subunit) (SF3b155)	SF3B1	0.005	1.01 ↑
P04216	Thy-1 membrane glycoprotein (CDw90)	THY1	0.005	1.01 ↑
O43396	Thioredoxin-like protein 1 (32 kDa thioredoxin-related protein)	TXNL1	0.004	1.01 ↑
Q15459	Splicing factor 3A subunit 1 (SF3a120)	SF3A1	0.004	1.02 ↑
A0A0U1RRL7	Protein MMP24OS (MMP24 opposite strand)	MMPOS	0.003	1.02 ↑
P38646	Stress-70 protein, mitochondrial (75 kDa glucose-regulated protein) (GRP-75)	GRP75	0.003	1.01 ↑
Q13501	Sequestosome-1 (Ubiquitin-binding protein p62)	SQSTM	0.003	1.04 ↑
P40925	Malate dehydrogenase, cytoplasmic	MDHC	0.002	1.01 ↑
P37837	Transaldolase	TALDO	0.002	1.02 ↑
P02545	Prelamin-A/C [Cleaved into: Lamin-A/C (70 kDa lamin) (Renal carcinoma antigen NY-REN-32)]	LMNA	0.002	1.01 ↑
P48637	Glutathione synthetase (GSH synthetase) (GSH-S)	GSHB	0.002	1.03 ↑
P05413	Fatty acid-binding protein, heart (Fatty acid-binding protein 3) (Heart-type fatty acid-binding protein) (H-FABP)	FABPH	0.001	1.03 ↑
P40926	Malate dehydrogenase, mitochondrial	MDHM	0.001	1.01 ↑
P08195	4F2 cell-surface antigen heavy chain (4F2hc)	4F2	0.0007	1.04 ↑
O95817	BAG family molecular chaperone regulator 3 (BAG-3)	BAG3	0.0004	1.04 ↑
Q8N6T3	ADP-ribosylation factor GTPase-activating protein 1 (ARF GAP 1)	ARFG1	0.0003	1.03 ↑

Table S6. List of altered proteins in U87MG cells versus Control, after treatment with 0.5 µM ST1926 for 2 hours. A total of 115 proteins with significant changes (61 downregulated (↓) and 54 upregulated (↑)) were identified in U87MG cells with $p < 0.05$.

Protein IDs	Protein Names	Gene IDs	*p*-value	Fold Change
P06737	Glycogen phosphorylase	PYGL	0.0002	0.98 ↓
P56192	Methionine--tRNA ligase	MARS1	0.0003	0.99 ↓
P12956	X-ray repair cross-complementing protein 6	XRCC6	0.0004	0.99 ↓
P46940	Ras GTPase-activating-like protein IQGAP1 (p195)	IQGAP1 KIAA0051	0.0004	0.99 ↓
P04040	Catalase	CAT	0.0005	0.98 ↓
P09382	Galectin-1 (Gal-1)	LGALS1	0.0005	0.98 ↓
P29966	Myristoylated alanine-rich C-kinase substrate	MARCKS	0.0008	0.98 ↓
P07196	Neurofilament light polypeptide (NF-L)	NEFL	0.001	0.98 ↓
P14625	Endoplasmin	HSP90B1	0.002	0.99 ↓
O75369	Filamin-B (FLN-B)	FLNB	0.003	0.99 ↓
Q8NBS9	Thioredoxin domain-containing protein 5	TXNDC5	0.004	0.99 ↓
P49327	Fatty acid synthase	FASN/ FAS	0.004	0.99 ↓
P13667	Protein disulfide-isomerase A4	PDIA4	0.005	0.98 ↓
O00469	Procollagen-lysine,2-oxoglutarate 5-dioxygenase 2	PLOD2	0.005	0.99 ↓
P27797	Calreticulin (CRP55) (Calregulin)	CALR	0.006	0.99 ↓
Q58FF8	Putative heat shock protein HSP 90-beta	HSP90AB2 P	0.007	0.98 ↓
P05455	Lupus La protein	SSB	0.008	0.98 ↓
P55735	Protein SEC13 homolog	SEC13	0.009	0.99 ↓
P39748	Flap endonuclease 1 (FEN-1)	FEN1 RAD2	0.009	0.98 ↓
P04899	Guanine nucleotide-binding protein G(i) subunit alpha-2	GNAI2 GNAI2B	0.01	0.98 ↓
P61978	Heterogeneous nuclear ribonucleoprotein K (hnRNP K)	HNRNPK HNRPK	0.01	0.99 ↓
P13010	X-ray repair cross-complementing protein 5	XRCC5	0.01	0.99 ↓
P22234	Multifunctional protein ADE2	PAICS ADE2	0.01	0.99 ↓
P49915	GMP synthase	GMPS	0.01	0.99 ↓
Q9Y2W1	Thyroid hormone receptor-associated protein 3	THRAP3	0.02	0.98 ↓
O15427	Monocarboxylate transporter 4 (MCT 4)	SLC16A3 MCT4	0.02	0.98 ↓
Q00610	Clathrin heavy chain 1	CLTC	0.02	0.99 ↓
P29401	Transketolase (TK)	TKT	0.02	0.98 ↓

Protein IDs	Protein Names	Gene IDs	*p*-value	Fold Change
P63208	S-phase kinase-associated protein 1	SKP1	0.02	0.99 ↓
Q86UP2	Kinectin	KTN1	0.02	0.99 ↓
P26583	High mobility group protein B2 (HMG-2)	HMGB2 HMG2	0.02	0.97 ↓
P07237	Protein disulfide-isomerase (PDI)	PDI	0.02	0.99 ↓
Q7KZF4	Staphylococcal nuclease domain-containing protein 1	SND1 TDRD11	0.02	0.99 ↓
P08621	U1 small nuclear ribonucleoprotein 70 kDa	SNRNP70	0.02	0.99 ↓
Q16531	DNA damage-binding protein 1	DDB1	0.03	0.99 ↓
P43307	Translocon-associated protein subunit alpha (TRAP-alpha)	SSR1 TRAPA	0.03	0.99↓
O94906	Pre-mRNA-processing factor 6	PRPF6	0.03	0.99 ↓
P30419	Glycylpeptide N-tetradecanoyltransferase 1	NMT1	0.03	0.96 ↓
P49321	Nuclear autoantigenic sperm protein (NASP)	NASP	0.03	0.99 ↓
P84077	ADP-ribosylation factor 1	ARF1	0.03	0.99 ↓
P50454	Serpin family H member 1	SERPINH1	0.04	0.99 ↓
Q13442	28 kDa heat- and acid-stable phosphoprotein (PDGF-associated protein) (PAP)	PDAP1 HASPP28	0.04	0.98 ↓
P62873	Guanine nucleotide-binding protein G(I)/G(S)/G(T) subunit beta-1 (Transducin beta chain 1)	GNB1	0.04	0.98 ↓
P09543	2',3'-cyclic-nucleotide 3'-phosphodiesterase	CNP	0.04	0.98 ↓
P31040	Succinate dehydrogenase [ubiquinone] flavoprotein subunit	SDHA	0.04	0.98 ↓
O75947	ATP synthase subunit d	ATP5PD	0.04	0.96 ↓
P27816	Microtubule-associated protein 4 (MAP-4)	MAP4	0.04	0.98 ↓
P27824	Calnexin	CANX	0.04	0.99 ↓
O43852	Calumenin	CALU	0.04	0.99 ↓
P39687	Acidic leucine-rich nuclear phosphoprotein 32 family member A	ANP32A	0.04	0.98 ↓
P09874	Poly [ADP-ribose] polymerase 1 (PARP-1)	PARP1	0.04	0.99 ↓
P11047	Laminin subunit gamma-1	LAMB2	0.04	0.98 ↓
P50990	T-complex protein 1 subunit theta (TCP-1-theta)	CCT8 CCTQ	0.04	0.99 ↓
P11717	Cation-independent mannose-6-phosphate receptor (CI Man-6-P receptor)	IGF2R MPRI	0.04	0.99 ↓
P14866	Heterogeneous nuclear ribonucleoprotein L (hnRNP L)	HNRNPL HNRPL	0.04	0.99 ↓
O75390	Citrate synthase	CS	0.040	0.99 ↓
O95573	Long-chain-fatty-acid--CoA ligase 3	ACSL3	0.04	0.98 ↓

Protein IDs	Protein Names	Gene IDs	*p*-value	Fold Change
P07910	Heterogeneous nuclear ribonucleoproteins C1/C2	HNRNPC	0.04	0.99 ↓
P28066	Proteasome subunit alpha type-5	PSMA5	0.04	0.99 ↓
P63241	Eukaryotic translation initiation factor 5A-1 (eIF-5A-1)	EIF5A	0.04	0.99 ↓
Q02809	Procollagen-lysine,2-oxoglutarate 5-dioxygenase 1	PLOD1<br>LLH	0.04	0.98 ↓
Q96RP9	Elongation factor G	GFM1	0.04	1.02 ↑
Q01081	Splicing factor U2AF 35 kDa subunit	U2AF1	0.04	1.02 ↑
P15880	40S ribosomal protein S2	RPS2	0.04	1.01 ↑
Q96T88	E3 ubiquitin-protein ligase UHRF1	UHRF1	0.04	1.02 ↑
P04792	Heat shock protein beta-1 (HspB1)	HSPB1	0.04	1.02 ↑
P60842	Eukaryotic initiation factor 4A-I (eIF-4A-I)	EIF4A1	0.04	1.01 ↑
P18124	60S ribosomal protein L7	RPL7	0.04	1.03 ↑
P68366	Tubulin alpha-4A chain	TUBA4A	0.04	1.03 ↑
Q01844	RNA-binding protein EWS	EWSR1	0.04	1.01 ↑
Q07955	Serine/arginine-rich splicing factor 1	SRSF1	0.04	1.02 ↑
Q8N1F7	Nuclear pore complex protein Nup93	NUP93	0.04	1.01 ↑
P24928	DNA-directed RNA polymerase II subunit RPB1 (RNA polymerase II subunit B1)	POLR2A<br>POLR2	0.04	1.01 ↑
P05386	60S acidic ribosomal protein P1	RPLP1	0.04	1.01 ↑
P42677	40S ribosomal protein S27	RPS27	0.03	1.02 ↑
P62241	40S ribosomal protein S8	RPS8	0.03	1.01 ↑
P17655	Calpain-2 catalytic subunit	CAPN2	0.03	1.02 ↑
Q13151	Heterogeneous nuclear ribonucleoprotein A0 (hnRNP A0)	HNRNPA0<br>HNRPA0	0.03	1.02 ↑
P41567	Eukaryotic translation initiation factor 1 (eIF1)	EIF1	0.03	1.08 ↑
Q16186	Proteasomal ubiquitin receptor ADRM1	ADRM1	0.03	1.04 ↑
P26038	Moesin (Membrane-organizing extension spike protein)	MSN	0.03	1.01 ↑
Q13200	26S proteasome non-ATPase regulatory subunit 2	PSMD2<br>TRAP2	0.03	1.01 ↑
P02786	Transferrin receptor protein 1 (TfR1)	TFRC	0.03	1.02 ↑
O94925	Glutaminase kidney isoform (GLS)/ (K-glutaminase)	GLS<br>GLS1	0.03	1.02 ↑
Q9H0A0	RNA cytidine acetyltransferase	NAT10	0.02	1.02 ↑
Q04637	Eukaryotic translation initiation factor 4 gamma 1 (eIF-4-gamma 1)	EIF4G1	0.02	1.02 ↑
Q16543	Hsp90 co-chaperone Cdc37	CDC37	0.02	1.01 ↑
O43396	Thioredoxin-like protein 1	TXNL1	0.02	1.02 ↑

Protein IDs	Protein Names	Gene IDs	*p*-value	Fold Change
P10644	cAMP-dependent protein kinase type I-alpha regulatory subunit	PRKAR1A	0.02	1.02 ↑
P40222	Alpha-taxilin	TXLNA	0.02	1.01 ↑
Q9NR30	Nucleolar RNA helicase 2 (DEAD box protein 21)	DDX21	0.01	1.02 ↑
Q5JTH9	RRP12-like protein	RRP12	0.01	1.03 ↑
P46781	40S ribosomal protein S9	RPS9	0.01	1.01 ↑
Q02878	60S ribosomal protein L6	RPL6	0.01	1.02 ↑
Q9NYF8	Bcl-2-associated transcription factor 1 (Btf)	BCLAF1	0.009	1.02 ↑
P31937	3-hydroxyisobutyrate dehydrogenase	HIBADH	0.008	1.01 ↑
P61221	ATP-binding cassette sub-family E member 1	ABCE1	0.005	1.01 ↑
Q13501	Sequestosome-1 (EBI3-associated protein of 60 kDa) (EBIAP) (p60)	SQSTM1	0.005	1.05 ↑
Q08J23	RNA cytosine C(5)-methyltransferase NSUN2	NSUN2	0.005	1.04 ↑
Q92598	Heat shock protein 105 kDa	HSPH1 HSP105	0.005	1.01 ↑
Q8TDN6	Ribosome biogenesis protein BRX1 homolog	BRIX1	0.005	1.02 ↑
Q9UMX0	Ubiquilin-1	UBQLN1	0.005	1.03 ↑
Q07960	Rho GTPase-activating protein 1	ARHGAP1	0.005	1.02 ↑
Q02543	60S ribosomal protein L18a	RPL18A	0.005	1.02 ↑
Q15942	Zyxin (Zyxin-2)	ZYX	0.004	1.07 ↑
P35268	60S ribosomal protein L22	RPL22	0.004	1.01 ↑
O15355	Protein phosphatase 1G	PPM1G	0.004	1.01 ↑
P50914	60S ribosomal protein L14	RPL14	0.004	1.02 ↑
P46060	Ran GTPase-activating protein 1 (RanGAP1)	RANGAP1	0.003	1.02 ↑
P17844	Probable ATP-dependent RNA helicase DDX5 (DEAD box protein 5)	DDX5	0.002	1.02 ↑
Q92945	Far upstream element-binding protein 2 (FUSE-binding protein 2)	KHSRP FUBP2	0.002	1.01 ↑
Q86TG7	Retrotransposon-derived protein PEG10	PEG10	0.001	1.04 ↑
P09493	Tropomyosin alpha-1 chain	TPM1	0.0007	1.04 ↑
P46087	Probable 28S rRNA (cytosine(4447)-C (5))-methyltransferase	NOP2	0.0007	1.02 ↑
P52292	Importin subunit alpha-1	KPNA	0.0002	1.03 ↑

Table S7. List of altered proteins in U87MG cells versus Control, after treatment with 0.5 µM ST1926 for 24 hours. A total of 167 proteins with significant changes (79 downregulated (↓) and 88 upregulated (↑)) were identified in U87MG with $p < 0.05$.

Protein IDs	Protein Names	Gene IDs	*p*-value	Fold Change
P07196	Neurofilament light polypeptide (NF-L)	NEFL	0.00001	0.97 ↓
Q9P2E9	Ribosome-binding protein 1	RRBP1	0.00004	0.93 ↓
Q58FF8	Putative heat shock protein HSP 90-beta	HSP90AB2P	0.0003	0.95 ↓
P14625	Endoplasmin	HSP90B1	0.0008	0.99 ↓
Q86UP2	Kinectin	KTN1	0.0009	0.98 ↓
Q08380	Galectin-3-binding protein (Gal-3BP)	LGALS3BP	0.0009	0.96 ↓
P12109	Collagen alpha-1(VI) chain	COL6A1	0.001	0.96 ↓
P04899	Guanine nucleotide-binding protein G(i) subunit alpha-2	GNAI2 GNAI2B	0.001	0.98 ↓
P46013	Proliferation marker protein Ki-67	MKI67	0.001	0.95 ↓
P08621	U1 small nuclear ribonucleoprotein 70 kDa	SNRNP70	0.002	0.99 ↓
P63208	S-phase kinase-associated protein 1	SKP1	0.002	0.97 ↓
P11717	Cation-independent mannose-6-phosphate receptor (CI Man-6-P receptor)	IGF2R MPRI	0.002	0.99 ↓
Q13443	A disintegrin and a metalloprotease 9	ADAM9	0.002	0.94 ↓
Q8NBS9	Thioredoxin domain-containing protein 5	TXNDC5	0.003	0.98 ↓
P13667	Protein disulfide-isomerase A4	PDIA4	0.003	0.98 ↓
P51114	Fragile X mental retardation syndrome-related protein 1	FXR1	0.004	0.98 ↓
Q02809	Procollagen-lysine,2-oxoglutarate 5-dioxygenase 1	PLOD1 LLH	0.004	0.96 ↓
P30101	protein disulfide isomerase family A member 3	PDIA3	0.004	0.99 ↓
Q7KZF4	Staphylococcal nuclease domain-containing protein 1	SND1 TDRD11	0.005	0.98 ↓
Q92896	Golgi apparatus protein 1 (CFR-1)	GLG1	0.005	0.97 ↓
P56192	Methionine--tRNA ligase	MARS1	0.005	0.99 ↓
P28066	Proteasome subunit alpha type-5	PSMA5	0.006	0.99 ↓
P05455	Lupus La protein	SSB	0.006	0.98 ↓
P52292	Importin subunit alpha-1	KPNA	0.006	0.99 ↓
P30876	RNA polymerase II subunit RPB2	POLR2B	0.007	0.98 ↓
P49327	Fatty acid synthase	FASN/ FAS	0.008	0.99 ↓
Q13263	Transcription intermediary factor 1-beta	TIF1B	0.008	0.98 ↓
P39656	Dolichyl-diphosphooligosaccharide--protein glycosyltransferase 48 kDa subunit	OST48	0.009	0.99 ↓

Protein IDs	Protein Names	Gene IDs	*p*-value	Fold Change
P40763	Signal transducer and activator of transcription 3	STAT3 APRF	0.009	0.98 ↓
O60313	Dynamin-like 120 kDa protein, mitochondrial	OPA1	0.01	0.97 ↓
O15427	Monocarboxylate transporter 4 (MCT 4)	SLC16A3 MCT4	0.01	0.98 ↓
P31946	14-3-3 protein beta/alpha (Protein 1054) (Protein kinase C inhibitor protein 1) (KCIP-1)	YWHAB	0.01	0.99 ↓
P25787	Proteasome subunit alpha type-2 (PSA2)	PSMA2	0.01	0.99 ↓
P29401	Transketolase (TK)	TKT	0.01	0.99 ↓
P12956	X-ray repair cross-complementing protein 6	XRCC6	0.01	0.99 ↓
Q9H3P7	Golgi resident protein GCP60	ACBD3	0.01	0.98 ↓
O00264	Membrane-associated progesterone receptor component 1 (mPR)	PGRMC1	0.01	0.98 ↓
P13987	CD59 glycoprotein	CD59	0.01	0.99 ↓
Q2TAY7	WD40 repeat-containing protein SMU1	SMU1	0.02	0.98 ↓
P35606	Coatomer subunit beta'	COPB2	0.02	0.98 ↓
P12277	Creatine kinase B-type	CKB	0.02	0.98 ↓
O14617	AP-3 complex subunit delta-1	AP3D1	0.02	0.98 ↓
P13010	X-ray repair cross-complementing protein 5	XRCC5	0.02	0.99 ↓
Q8WX93	Palladin	PALLD	0.02	0.98 ↓
P09382	Galectin-1 (Gal-1)	LGALS1	0.02	0.99 ↓
P63092	Guanine nucleotide-binding protein G(s) subunit alpha isoforms short	GNAS GNAS1	0.02	0.98 ↓
P12004	Proliferating cell nuclear antigen	PCNA	0.02	0.98 ↓
Q16531	DNA damage-binding protein 1	DDB1	0.02	0.99 ↓
P16278	Beta-galactosidase	GLB1	0.02	0.98 ↓
Q14980	Nuclear mitotic apparatus protein 1	NUMA1	0.03	0.97 ↓
P13804	Electron transfer flavoprotein subunit alpha, mitochondrial	ETFA	0.03	0.99 ↓
Q1KMD3	Heterogeneous nuclear ribonucleoprotein U-like protein 2	HNRNPUL 2	0.03	0.99 ↓
P11310	Medium-chain specific acyl-CoA dehydrogenase, mitochondrial	ACADM	0.03	0.97 ↓
Q15019	Septin-2	SEPTIN2	0.03	0.99 ↓
P13674	Prolyl 4-hydroxylase subunit alpha-1	P4HA1	0.03	0.99 ↓
P83916	Chromobox protein homolog 1 (HP1Hsbeta)	CBX1	0.03	0.99 ↓
P52788	Spermine synthase (SPMSY)	SMS	0.03	0.99 ↓
O14980	Exportin-1 (Exp1)	XPO1	0.03	0.99 ↓

Protein IDs	Protein Names	Gene IDs	*p*-value	Fold Change
P43307	Translocon-associated protein subunit alpha (TRAP-alpha)	SSR1 TRAPA	0.03	0.99 ↓
P39687	Acidic leucine-rich nuclear phosphoprotein 32 family member A	ANP32A	0.03	0.98 ↓
Q96HE7	ERO1-like protein alpha	ERO1A	0.04	0.98 ↓
Q15155	Nodal modulator 1	NOMO1	0.04	0.99 ↓
Q99623	Prohibitin-2	PHB2	0.04	0.99 ↓
Q92499	ATP-dependent RNA helicase DDX1	DDX1	0.04	0.99 ↓
P30419	Glycylpeptide N-tetradecanoyltransferase 1	NMT1	0.04	0.99 ↓
Q9Y5L0	Transportin-3	TNPO3	0.04	0.98 ↓
P07237	Protein disulfide-isomerase (PDI)	PDI	0.04	0.99 ↓
Q9UBG0	C-type mannose receptor 2	MRC2	0.04	0.96 ↓
P48735	Isocitrate dehydrogenase [NADP], mitochondrial	IDH2	0.04	0.97 ↓
P11388	DNA topoisomerase 2-alpha	TOP2A TOP2	0.04	0.96 ↓
O00469	Procollagen-lysine,2-oxoglutarate 5-dioxygenase 2	PLOD2	0.04	0.98 ↓
P49748	Very long-chain specific acyl-CoA dehydrogenase	ACADVL	0.04	0.99 ↓
P60174	Triosephosphate isomerase (TIM)	TPI1/TPI	0.04	0.99 ↓
P84077	ADP-ribosylation factor 1	ARF1	0.04	0.99 ↓
P35221	Catenin alpha-1 (Alpha E-catenin)	CTNNA1	0.04	0.99 ↓
P78347	General transcription factor II-I (GTFII-I)	GTF2I	0.04	0.98 ↓
Q15293	Reticulocalbin-1	RCN1	0.04	0.99 ↓
P31689	DnaJ homolog subfamily A member 1 (DnaJ protein homolog 2)	DNAJA1 DNAJ2	0.04	0.96 ↓
P11047	Laminin subunit gamma-1 (Laminin B2 chain)	LAMB2	0.04	0.91 ↓
P04083	Annexin A1 (Annexin I)	ANXA1	0.04	1.01 ↑
P62266	40S ribosomal protein S23	RPS23	0.04	1.08 ↑
Q99613	Eukaryotic translation initiation factor 3 subunit C (eIF3c)	EIF3C EIF3S8	0.04	1.02 ↑
Q02952	A-kinase anchor protein 12 (AKAP-12)	AKAP12	0.04	1.01 ↑
P15880	40S ribosomal protein S2	RPS2	0.04	1.02 ↑
Q13595	Transformer-2 protein homolog alpha	TRA2A	0.04	1.06 ↑
P23381	Tryptophan--tRNA ligase	WARS1	0.04	1.02 ↑
P35268	60S ribosomal protein L22	RPL22	0.04	1.00 ↑
Q9UNZ2	NSFL1 cofactor p47	NSFL1C	0.04	1.09 ↑
P63220	40S ribosomal protein S21	RPS21	0.04	1.01 ↑

Protein IDs	Protein Names	Gene IDs	*p*-value	Fold Change
Q16543	Hsp90 co-chaperone Cdc37	CDC37	0.04	1.01 ↑
P23246	Splicing factor, proline- and glutamine-rich	SFPQ	0.04	1.02 ↑
P62280	40S ribosomal protein S11	RPS11	0.04	1.02 ↑
Q04637	Eukaryotic translation initiation factor 4 gamma 1 (eIF-4-gamma 1)	EIF4G1	0.04	1.01 ↑
Q05682	Caldesmon	CALD1	0.04	1.02 ↑
A0A0U1	Protein MMP24OS	MMP24OS	0.04	1.02 ↑
O00487	26S proteasome non-ATPase regulatory subunit 14	PSMD14	0.03	1.02 ↑
Q9Y5L4	Mitochondrial import inner membrane translocase subunit Tim13	TIMM13	0.03	1.03 ↑
Q8WUM4	Programmed cell death 6-interacting protein	PDCD6IP	0.03	1.02 ↑
P26373	60S ribosomal protein L13	RPL13	0.03	1.04 ↑
P09651	Heterogeneous nuclear ribonucleoprotein A1	HNRNPA1	0.03	1.01 ↑
P42677	40S ribosomal protein S27	RPS27	0.03	1.03 ↑
P40222	Alpha-taxilin	TXLNA	0.03	1.02 ↑
P41567	Eukaryotic translation initiation factor 1 (eIF1)	EIF1	0.03	1.09 ↑
P62277	40S ribosomal protein S13	RPS13	0.03	1.02 ↑
P08195	4F2 cell-surface antigen heavy chain (4F2hc)	SLC3A2	0.03	1.02 ↑
Q9HAV4	Exportin-5	XPO5	0.03	1.02 ↑
Q5JTH9	RRP12-like protein	RRP12	0.03	1.03 ↑
Q14011	Cold-inducible RNA-binding protein	CIRBP	0.03	1.02 ↑
Q9NYF8	Bcl-2-associated transcription factor 1 (Btf)	BCLAF1	0.03	1.02 ↑
Q9BWM7	Sideroflexin-3	SFXN3	0.02	1.01 ↑
P50502	Hsc70-interacting protein (Hip)	ST13 FAM10A1	0.02	1.01 ↑
P22102	Trifunctional purine biosynthetic protein adenosine-3	GART	0.02	1.01 ↑
P18124	60S ribosomal protein L7	RPL7	0.02	1.03 ↑
Q02978	Mitochondrial 2-oxoglutarate/malate carrier protein	SLC25A11	0.02	1.01 ↑
P08865	40S ribosomal protein SA	RPSA	0.02	1.01 ↑
Q00325	Phosphate carrier protein	SLC25A6	0.02	1.02 ↑
P16989	Y-box-binding protein 3	YBX3	0.02	1.03 ↑
P16949	Stathmin	STMN1	0.02	1.05 ↑
P0DMV9	Heat shock 70 kDa protein 1B	HSPA1B	0.02	1.01 ↑
Q9P0L0	Vesicle-associated membrane protein-associated protein A (VAMP-A)	VAPA VAP33	0.02	1.01 ↑

Protein IDs	Protein Names	Gene IDs	*p*-value	Fold Change
P21333	Filamin-A (FLN-A)	FLNA/ FLN	0.02	1.01 ↑
P27635	60S ribosomal protein L10	RPL10	0.02	1.05 ↑
O60664	Perilipin-3	PLIN3	0.02	1.02 ↑
O14974	Protein phosphatase 1 regulatory subunit 12A	PPP1R12A	0.01	1.03 ↑
Q9NR30	Nucleolar RNA helicase 2 (DEAD box protein 21)	DDX21	0.01	1.02 ↑
P51858	Hepatoma-derived growth factor	HDGF	0.01	1.01 ↑
Q8TEX9	Importin-4	IPO4	0.01	1.02 ↑
P60842	Eukaryotic initiation factor 4A-I (eIF-4A-I)	EIF4A1	0.01	1.01 ↑
P16403	Histone H1.2	H1-2 HIST1H1C	0.01	1.02 ↑
P17844	Probable ATP-dependent RNA helicase DDX5 (DEAD box protein 5)	DDX5	0.01	1.02 ↑
P62241	40S ribosomal protein S8	RPS8	0.01	1.01 ↑
P62829	60S ribosomal protein L23	RPL23	0.01	1.03 ↑
P11940	Polyadenylate-binding protein 1	PABPC1	0.01	1.02 ↑
P68366	Tubulin alpha-4A chain	TUBA4A	0.01	1.03 ↑
Q16186	Proteasomal ubiquitin receptor ADRM1	ADRM1	0.01	1.03 ↑
Q07960	Rho GTPase-activating protein 1	ARHGAP1	0.01	1.02 ↑
Q01081	Splicing factor U2AF 35 kDa subunit	U2AF1	0.01	1.04 ↑
P19105	Myosin regulatory light chain 12A	MYL12A	0.009	1.01 ↑
P46781	40S ribosomal protein S9	RPS9	0.009	1.01 ↑
Q9UMX0	Ubiquilin-1	UBQLN1	0.008	1.03 ↑
P24928	DNA-directed RNA polymerase II subunit RPB1 (RNA polymerase II subunit B1)	POLR2A POLR2	0.008	1.02 ↑
Q9UQ80	Proliferation-associated protein 2G4	PA2G4	0.008	1.01 ↑
Q9Y5B9	FACT complex subunit SPT16	SUPT16H	0.007	1.01 ↑
P62913	60S ribosomal protein L11	RPL11	0.007	1.02 ↑
Q07955	Serine/arginine-rich splicing factor 1	SRSF1	0.006	1.02 ↑
P19338	Nucleolin	NCL	0.006	1.01 ↑
O75396	Vesicle-trafficking protein SEC22b	SEC22B	0.006	1.03 ↑
Q9H1E3	Nuclear ubiquitous casein and cyclin-dependent kinase substrate 1 (p21)	NUCKS1	0.006	1.03 ↑
P67809	Y-box-binding protein 1 (YB-1)	YBX1	0.005	1.03 ↑
P11142	Heat shock cognate 71 kDa protein	HSPA8 HSC70	0.005	1.01 ↑
P12236	Solute carrier family 25 member 6	SLC25A6	0.005	1.01 ↑
P31153	S-adenosylmethionine synthase isoform type-2	MAT2A	0.004	1.02 ↑

Protein IDs	Protein Names	Gene IDs	*p*-value	Fold Change
Q08J23	RNA cytosine C(5)-methyltransferase NSUN2	NSUN2	0.004	1.06 ↑
P05386	60S acidic ribosomal protein P1	RPLP1	0.003	1.02 ↑
P26641	Elongation factor 1-gamma (EF-1-gamma)	EEF1G EF1G	0.003	1.02 ↑
Q15050	Ribosome biogenesis regulatory protein homolog	RRS1	0.003	1.03 ↑
O15355	Protein phosphatase 1G	PPM1G	0.003	1.02 ↑
P56537	Eukaryotic translation initiation factor 6	EIF6	0.002	1.01 ↑
Q13151	Heterogeneous nuclear ribonucleoprotein A0 (hnRNP A0)	HNRNPA0 HNRPA0	0.002	1.03 ↑
P05387	60S acidic ribosomal protein P2	RPLP2	0.002	1.01 ↑
P09493	Tropomyosin alpha-1 chain	TPM1	0.001	1.04 ↑
Q15942	Zyxin (Zyxin-2)	ZYX	0.0008	1.10 ↑
Q13501	Sequestosome-1 (EBI3-associated protein of 60 kDa) (EBIAP) (p60)	SQSTM1	0.0007	1.07 ↑
P12268	Inosine-5'-monophosphate dehydrogenase 2 (IMP dehydrogenase 2) (IMPD 2)	IMPDH2 IMPD2	0.0005	1.02 ↑
Q8TDN6	Ribosome biogenesis protein BRX1 homolog	BRIX1	0.0004	1.03 ↑
Q92945	Far upstream element-binding protein 2 (FUSE-binding protein 2)	KHSRP FUBP2	0.0003	1.02 ↑
P46087	Probable 28S rRNA (cytosine (4447)-C (5))-methyltransferase	NOP2	0.00003	1.05 ↑

Table S8. List of altered proteins in U87MG cells versusControl, after treatment with 0.5 µM ST1926 for 48 hours. A total of 282 proteins with significant changes (125 downregulated (↓) and 157 upregulated (↑)) were identified in U87MG with $p < 0.05$.

Protein IDs	Protein Names	Gene IDs	*p*-value	Fold Change
P42704	Leucine-rich PPR motif-containing protein, mitochondrial (130 kDa leucine-rich protein) (LRP 130)	LPPRC	0.00008	0.99 ↓
P46013	Proliferation marker protein Ki-67 (Antigen Ki67)	KI67	0.0001	0.91 ↓
Q13185	Chromobox protein homolog 3 (HECH)	CBX3	0.0002	0.96 ↓
P07196	Neurofilament light polypeptide (NF-L)	NFL	0.0002	0.95 ↓
Q9P2E9	Ribosome-binding protein 1 (180 kDa ribosome receptor homolog) (RRp)	RRBP1	0.0003	0.93 ↓
Q9BY67	Cell adhesion molecule 1 (Immunoglobulin superfamily member 4) (IgSF4)	CADM1	0.0003	0.97 ↓
P11047	Laminin subunit gamma-1 (Laminin B2 chain)	LAMC1	0.0004	0.94 ↓
P26358	DNA (cytosine-5)-methyltransferase 1 (Dnmt1)	DNMT1	0.0004	0.91 ↓
O00469	Procollagen-lysine,2-oxoglutarate 5-dioxygenase 2 (Lysyl hydroxylase 2) (LH2)	PLOD2	0.0006	0.96 ↓
P14625	Endoplasmin (94 kDa glucose-regulated protein) (GRP-94)	ENPL	0.0006	0.99 ↓
P12956	X-ray repair cross-complementing protein 6	XRCC6	0.0006	0.99 ↓
O14979	Heterogeneous nuclear ribonucleoprotein D-like (hnRNP D-like)	HNRDL	0.0006	0.97 ↓
P06576	ATP synthase subunit beta, mitochondrial	ATPB	0.0007	0.99 ↓
P13667	Protein disulfide-isomerase A4	PDIA4	0.0007	0.98 ↓
P46940	Ras GTPase-activating-like protein IQGAP1 (p195)	IQGA1	0.0007	0.99 ↓
Q92896	Golgi apparatus protein 1 (CFR-1)	GSLG1	0.001	0.95 ↓
Q02809	Procollagen-lysine,2-oxoglutarate 5-dioxygenase 1 (Lysyl hydroxylase 1) (LH1)	PLOD1	0.001	0.95 ↓
Q09666	Neuroblast differentiation-associated protein AHNAK (Desmoyokin)	AHNK	0.001	0.99 ↓
P35613	Basigin (5F7) (Collagenase stimulatory factor) (Extracellular matrix metalloproteinase inducer) (EMMPRIN)	BASI	0.001	0.98 ↓
P11388	DNA topoisomerase 2-alpha	TOP2A	0.002	0.91 ↓
O95573	Fatty acid CoA ligase Acsl3 (Arachidonate--CoA ligase)	ACSL3	0.002	0.97 ↓
Q58FF8	Putative heat shock protein HSP 90-beta 2	H90B2	0.002	0.96 ↓
P26006	Integrin alpha-3 (CD49 antigen-like family member C) (FRP-2)	ITA3	0.002	0.98 ↓

**Protein IDs**	**Protein Names**	**Gene IDs**	***p*-value**	**Fold Change**
P04181	Ornithine aminotransferase, mitochondrial	OAT	0.002	0.95 ↓
P52292	Importin subunit alpha-1 (Karyopherin subunit alpha-2) (RAG cohort protein 1) (SRP1-alpha)	IMA1	0.002	0.96 ↓
P04899	Guanine nucleotide-binding protein G(i) subunit alpha-2	GNAI2	0.003	0.98 ↓
Q08380	Galectin-3-binding protein (Basement membrane autoantigen p105)	LG3BP	0.003	0.95 ↓
Q9Y224	RNA transcription, translation and transport factor protein (CLE7 homolog) (CLE) (hCLE)	RTRAF	0.004	0.99 ↓
P12004	Proliferating cell nuclear antigen (PCNA) (Cyclin)	PCNA	0.004	0.98 ↓
Q13263	Transcription intermediary factor 1-beta (TIF1-beta)	TIF1B	0.004	0.99 ↓
P30084	Enoyl-CoA hydratase, mitochondrial (mECH) (mECH1)	ECHM	0.004	0.98 ↓
O60313	Dynamin-like 120 kDa protein, mitochondrial (Optic atrophy protein 1)	OPA1	0.004	0.97 ↓
O60264	SWI/SNF-related matrix-associated actin-dependent regulator of chromatin subfamily A member 5	SMCA5	0.004	0.98 ↓
O75643	U5 small nuclear ribonucleoprotein 200 kDa helicase (Activating signal cointegrator 1 complex subunit 3-like 1) (BRR2 homolog)	U520	0.005	0.98 ↓
Q6DD88	Atlastin-3	ATLA3	0.005	0.98 ↓
O15427	Monocarboxylate transporter 4 (MCT 4)	MOT4	0.005	0.98 ↓
Q9Y2W1	Thyroid hormone receptor-associated protein 3 (BCLAF1 and THRAP3 family member 2)	TR150	0.006	0.98 ↓
P02786	Transferrin receptor protein 1 (TR) (TfR) (TfR1) (Trfr) (T9) (p90) (CD antigen CD71)	TFR1	0.006	0.98 ↓
P04216	Thy-1 membrane glycoprotein (CDw90) (Thy-1 antigen) (CD antigen CD90)	THY1	0.006	0.97 ↓
Q16790	Carbonic anhydrase 9	CAH9	0.006	0.97 ↓
P12277	Creatine kinase B-type (Brain creatine kinase) (B-CK)	KCRB	0.006	0.98 ↓
P16144	Integrin beta-4 (GP150) (CD antigen CD104)	ITB4	0.007	0.96 ↓
O75533	Splicing factor 3B subunit 1 (Pre-mRNA-splicing factor SF3b 155 kDa subunit)	SF3B1	0.007	0.99 ↓
P09382	Galectin-1 (Gal-1) (14 kDa laminin-binding protein) (HLBP14)	LEG1	0.008	0.99 ↓
Q8NBS9	Thioredoxin domain-containing protein 5	TXND5	0.008	0.98 ↓
Q96RP9	Elongation factor G, mitochondrial (EF-Gmt)	EFGM	0.009	0.97 ↓
Q9BZZ5	Apoptosis inhibitor 5 (API-5) (Antiapoptosis clone 11 protein)	API5	0.01	0.99 ↓

Protein IDs	Protein Names	Gene IDs	*p*-value	Fold Change
Q15907	Ras-related protein Rab-11B (GTP-binding protein YPT3)	RB11B	0.01	0.99 ↓
P50454	Serpin H1 (47 kDa heat shock protein) (Arsenic-transactivated protein 3) (AsTP3)	SERPH	0.01	0.99 ↓
O75475	PC4 and SFRS1-interacting protein (CLL-associated antigen KW-7) (Dense fine speckles 70 kDa protein) (DFS 70)	PSIP1	0.01	0.93 ↓
Q14980	Nuclear mitotic apparatus protein 1 (Nuclear matrix protein-22) (NMP-22)	NUMA1	0.01	0.97 ↓
P49327	Fatty acid synthase	FAS	0.01	0.99 ↓
P13674	Prolyl 4-hydroxylase subunit alpha-1 (4-PH alpha-1)	P4HA1	0.01	0.97 ↓
Q15717	ELAV-like protein 1 (Hu-antigen R) (HuR)	ELAV1	0.01	0.98 ↓
P49321	Nuclear autoantigenic sperm protein (NASP)	NASP	0.01	0.98 ↓
P43304	Glycerol-3-phosphate dehydrogenase, mitochondrial (GPD-M) (GPDH-M)	GPDM	0.01	0.97 ↓
O75534	Cold shock domain-containing protein E1 (N-ras upstream gene protein) (Protein UNR)	CSDE1	0.01	0.99 ↓
P51659	Peroxisomal multifunctional enzyme type 2 (MFE-2)	DHB4	0.01	0.98 ↓
Q32P28	Prolyl 3-hydroxylase 1	P3H1	0.01	0.98 ↓
P11717	Cation-independent mannose-6-phosphate receptor (CI Man-6-P receptor)	MPRI	0.01	0.98 ↓
Q14008	Cytoskeleton-associated protein 5 (Colonic and hepatic tumor overexpressed gene protein) (Ch-TOG)	CKAP5	0.01	0.98 ↓
Q9H3N1	Thioredoxin-related transmembrane protein 1 (Thioredoxin domain-containing protein 1) (Transmembrane Trx-related protein)	TMX1	0.01	0.98 ↓
P06748	Nucleophosmin (NPM)	NPM	0.01	0.98 ↓
P35221	Catenin alpha-1 (Alpha E-catenin)	CTNA1	0.01	0.98 ↓
Q14103	Heterogeneous nuclear ribonucleoprotein D0 (hnRNP D0)	HNRPD	0.01	0.99 ↓
P16278	Beta-galactosidase (Acid beta-galactosidase)	BGAL	0.02	0.98 ↓
Q96HE7	ERO1-like protein alpha (ERO1-L) (ERO1-L-alpha)	ERO1A	0.02	0.98 ↓
Q9H4G4	Golgi-associated plant pathogenesis-related protein 1 (GAPR-1) (Golgi-associated PR-1 protein) (Glioma pathogenesis-related protein 2) (GliPR 2)	GAPR1	0.02	0.97 ↓

Protein IDs	Protein Names	Gene IDs	*p*-value	Fold Change
Q15029	116 kDa U5 small nuclear ribonucleoprotein component (Elongation factor Tu GTP-binding domain-containing protein 2) (SNU114 homolog)	U5S1	0.02	0.99 ↓
P63208	S-phase kinase-associated protein 1 (Cyclin-A/CDK2-associated protein p19) (p19A)	SKP1	0.02	0.97 ↓
P62873	Guanine nucleotide-binding protein G(I)/G(S)/G(T) subunit beta-1 (Transducin beta chain 1)	GBB1	0.02	0.98 ↓
Q15393	Splicing factor 3B subunit 3	SF3B3	0.02	0.99 ↓
P13804	Electron transfer flavoprotein subunit alpha, mitochondrial (Alpha-ETF)	ETFA	0.02	0.97 ↓
P45877	Peptidyl-prolyl cis-trans isomerase C (PPIase C)	PPIC	0.02	0.98 ↓
Q16531	DNA damage-binding protein 1 (DDB p127 subunit)	DDB1	0.02	0.98 ↓
P30101	Protein disulfide-isomerase A3	PDIA3	0.02	0.98 ↓
P38159	RNA-binding motif protein, X chromosome (Glycoprotein p43)	RBMX	0.02	0.99 ↓
P61978	Heterogeneous nuclear ribonucleoprotein K (hnRNP K) (Transformation up-regulated nuclear protein) (TUNP)	HNRPK	0.02	0.98 ↓
P08670	Vimentin	VIME	0.02	0.99 ↓
O14980	Exportin-1 (Exp1) (Chromosome region maintenance 1 protein homolog)	XPO1	0.02	0.99 ↓
Q1KMD3	Heterogeneous nuclear ribonucleoprotein U-like protein 2 (Scaffold-attachment factor A2) (SAF-A2)	HNRL2	0.02	0.99 ↓
P30876	DNA-directed RNA polymerase II subunit RPB2	RPB2	0.02	0.95 ↓
P16615	Sarcoplasmic/endoplasmic reticulum calcium ATPase 2 (SERCA2) (SR Ca(2+)-ATPase 2)	AT2A2	0.02	0.97 ↓
P51398	Small ribosomal subunit protein mS29 (28S ribosomal protein S29, mitochondrial) (MRP-S29)	RT29	0.02	0.96 ↓
Q86UP2	Kinectin (CG-1 antigen) (Kinesin receptor)	KTN1	0.02	0.98 ↓
P07858	Cathepsin B (APP secretase) (APPS)	CATB	0.02	0.97 ↓
P61289	Proteasome activator complex subunit 3 (11S regulator complex subunit gamma) (REG-gamma)	PSME3	0.02	0.98 ↓
Q12769	Nuclear pore complex protein Nup160 (160 kDa nucleoporin) (Nucleoporin Nup160)	NU160	0.02	0.96 ↓
P43307	Translocon-associated protein subunit alpha (TRAP-alpha) (Signal sequence receptor subunit alpha) (SSR-alpha)	SSRA	0.02	0.98 ↓
Q9H0U4	Ras-related protein Rab-1B	RAB1B	0.02	0.98 ↓

Protein IDs	Protein Names	Gene IDs	*p*-value	Fold Change
P09661	U2 small nuclear ribonucleoprotein A' (U2 snRNP A')	RU2A	0.03	0.98 ↓
P63010	AP-2 complex subunit beta (AP105B)	AP2B1	0.03	0.99 ↓
P07910	Heterogeneous nuclear ribonucleoproteins C1/C2 (hnRNP C1/C2)	HNRPC	0.03	0.98 ↓
P62191	26S proteasome regulatory subunit 4 (P26s4)	PRS4	0.03	0.98 ↓
P62805	Histone H4	H4	0.03	0.99 ↓
P61586	Transforming protein RhoA (Rho cDNA clone 12) (h12)	RHOA	0.03	0.98 ↓
P07900	Heat shock protein HSP 90-alpha (Heat shock 86 kDa) (HSP 86) (HSP86)	HS90A	0.03	0.99 ↓
P16401	Histone H1.5 (Histone H1a) (Histone H1b) (Histone H1s-3)	H15	0.03	0.98 ↓
Q92499	ATP-dependent RNA helicase DDX1 (DEAD box protein 1) (DEAD box protein retinoblastoma) (DBP-RB)	DDX1	0.03	0.99 ↓
P05023	Sodium/potassium-transporting ATPase subunit alpha-1 (Na(+)/K(+) ATPase alpha-1 subunit)	AT1A1	0.03	0.99 ↓
P12109	Collagen alpha-1(VI) chain	CO6A1	0.03	0.96 ↓
P78527	DNA-dependent protein kinase catalytic subunit (DNA-PK catalytic subunit) (DNA-PKcs) (DNPK1) (p460)	PRKDC	0.03	0.99 ↓
P51114	RNA-binding protein FXR1 (FMR1 autosomal homolog 1) (hFXR1p)	FXR1	0.03	0.98 ↓
P08238	Heat shock protein HSP 90-beta (HSP 90) (Heat shock 84 kDa) (HSP 84) (HSP84)	HS90B	0.03	0.99 ↓
Q14978	Nucleolar and coiled-body phosphoprotein 1 (140 kDa nucleolar phosphoprotein) (Nopp140) (Hepatitis C virus NS5A-transactivated protein 13)	NOLC1	0.03	0.97 ↓
P62760	Visinin-like protein 1 (VILIP) (VLP-1) (Hippocalcin-like protein 3) (HLP3)	VISL1	0.04	0.98 ↓
P48681	Nestin	NEST	0.04	0.99 ↓
P54886	Delta-1-pyrroline-5-carboxylate synthase (P5CS)	P5CS	0.04	0.98 ↓
P55084	Trifunctional enzyme subunit beta, mitochondrial (TP-beta)	ECHB	0.04	0.99 ↓
P0DP25	Calmodulin-3	CALM3	0.04	0.99 ↓
P17844	Probable ATP-dependent RNA helicase DDX5 (DEAD box protein 5) (RNA helicase p68)	DDX5	0.04	0.99 ↓
Q68CZ2	Tensin-3 (Tensin-like SH2 domain-containing protein 1) (Tumor endothelial marker 6)	TENS3	0.04	0.96 ↓
Q9NZM1	Myoferlin (Fer-1-like protein 3)	MYOF	0.04	0.98 ↓

Protein IDs	Protein Names	Gene IDs	*p*-value	Fold Change
Q9UBB4	Ataxin-10 (Brain protein E46 homolog) (Spinocerebellar ataxia type 10 protein)	ATX10	0.04	0.97 ↓
Q12797	Aspartyl/asparaginyl beta-hydroxylase (Aspartate beta-hydroxylase)	ASPH	0.04	0.99 ↓
Q15233	Non-POU domain-containing octamer-binding protein (NonO protein)	NONO	0.04	0.98 ↓
P39656	Dolichyl-diphosphooligosaccharide--protein glycosyltransferase 48 kDa subunit (DDOST 48 kDa subunit) (Oligosaccharyl transferase 48 kDa subunit)	OST48	0.04	0.99 ↓
Q15084	Protein disulfide-isomerase A6 (Endoplasmic reticulum protein 5) (ER protein 5) (ERp5) (Protein disulfide isomerase P5) (Thioredoxin domain-containing protein 7)	PDIA6	0.04	0.99 ↓
Q9Y2J8	Protein-arginine deiminase type-2 (PAD-H19) (Peptidylarginine deiminase II) (Protein-arginine deiminase type II)	PADI2	0.04	0.99 ↓
Q15293	Reticulocalbin-1	RCN1	0.04	0.99 ↓
Q07065	Cytoskeleton-associated protein 4 (63-kDa cytoskeleton-linking membrane protein) (Climp-63) (p63)	CKAP4	0.04	0.99 ↓
Q9BSJ8	Extended synaptotagmin-1 (E-Syt1) (Membrane-bound C2 domain-containing protein)	ESYT1	0.04	0.99 ↓
P49792	E3 SUMO-protein ligase RanBP2 (358 kDa nucleoporin) (Nuclear pore complex protein Nup358) (Nucleoporin Nup358) (Ran-binding protein 2) (RanBP2) (p270)	RBP2	0.04	0.90 ↓
P08559	Pyruvate dehydrogenase E1 component subunit alpha, somatic form, mitochondrial (PDHE1-A type I)	ODPA	0.04	0.98 ↓
O00231	26S proteasome non-ATPase regulatory subunit 11 (26S proteasome regulatory subunit RPN6) (26S proteasome regulatory subunit S9) (26S proteasome regulatory subunit p44.5)	PSD11	0.04	0.99 ↓
O15260	Surfeit locus protein 4	SURF4	0.04	0.99 ↓
P08708	Small ribosomal subunit protein eS17 (40S ribosomal protein S17)	RS17	0.04	1.03 ↑
P68036	Ubiquitin-conjugating enzyme E2 L3 (E2 ubiquitin-conjugating enzyme L3)	UB2L3	0.04	1.03 ↑
P23588	Eukaryotic translation initiation factor 4B (eIF-4B)	IF4B	0.04	1.03 ↑
Q99615	DnaJ homolog subfamily C member 7 (Tetratricopeptide repeat protein 2)	DNJC7	0.04	1.02 ↑

Protein IDs	Protein Names	Gene IDs	*p*-value	Fold Change
Q13435	Splicing factor 3B subunit 2 (Pre-mRNA-splicing factor SF3b 145 kDa subunit) (SF3b145)	SF3B2	0.04	1.01 ↑
Q02790	Peptidyl-prolyl cis-trans isomerase FKBP4 (PPIase FKBP4) (51 kDa FK506-binding protein) (FKBP51)	FKBP4	0.04	1.01 ↑
P53004	Biliverdin reductase A (BVR A) (Biliverdin-IX alpha-reductase)	BIEA	0.04	1.02 ↑
Q9Y265	RuvB-like 1 (49 kDa TATA box-binding protein-interacting protein)	RUVB1	0.04	1.01 ↑
P83731	Large ribosomal subunit protein eL24 (60S ribosomal protein L24)	RL24	0.04	1.10 ↑
P14314	Glucosidase 2 subunit beta (80K-H protein)	GLU2B	0.04	1.01 ↑
Q96C19	EF-hand domain-containing protein D2 (Swiprosin-1)	EFHD2	0.04	1.02 ↑
P47895	Retinaldehyde dehydrogenase 3 (RALDH-3)	AL1A3	0.04	1.04 ↑
P17174	Aspartate aminotransferase, cytoplasmic (cAspAT)	AATC	0.04	1.02 ↑
Q02543	Large ribosomal subunit protein eL20 (60S ribosomal protein L18a)	RL18A	0.04	1.01 ↑
P13797	Plastin-3 (T-plastin)	PLST	0.04	1.01 ↑
P62249	Small ribosomal subunit protein uS9 (40S ribosomal protein S16)	RS16	0.04	1.02 ↑
Q13155	Aminoacyl tRNA synthase complex-interacting multifunctional protein 2 (Multisynthase complex auxiliary component p38) (Protein JTV-1)	AIMP2	0.04	1.02 ↑
O60664	Perilipin-3 (47 kDa mannose 6-phosphate receptor-binding protein)	PLIN3	0.04	1.03 ↑
Q99798	Aconitate hydratase, mitochondrial (Aconitase) (Citrate hydro-lyase)	ACON	0.04	1.01 ↑
P04792	Heat shock protein beta-1 (HspB1) (28 kDa heat shock protein)	HSPB1	0.04	1.01 ↑
P61353	Large ribosomal subunit protein eL27 (60S ribosomal protein L27)	RL27	0.04	1.02 ↑
Q06830	Peroxiredoxin-1 (Natural killer cell-enhancing factor A) (NKEF-A)	PRDX1	0.04	1.01 ↑
P55884	Eukaryotic translation initiation factor 3 subunit B (eIF3b)	EIF3B	0.04	1.01 ↑
P46060	Ran GTPase-activating protein 1 (RanGAP1)	RAGP1	0.04	1.01 ↑
Q07960	Rho GTPase-activating protein 1 (CDC42 GTPase-activating protein)	RHG01	0.04	1.02 ↑
Q14192	Four and a half LIM domains protein 2 (FHL-2) (LIM domain protein DRAL) (Skeletal muscle LIM-protein 3) (SLIM-3)	FHL2	0.03	1.08 ↑

Protein IDs	Protein Names	Gene IDs	*p*-value	Fold Change
P04075	Fructose-bisphosphate aldolase A (Lung cancer antigen NY-LU-1) (Muscle-type aldolase)	ALDOA	0.03	1.01 ↑
Q9BRJ6	Uncharacterized protein C7orf50	CG050	0.03	1.01 ↑
Q9NRX4	14 kDa phosphohistidine phosphatase (Phosphohistidine phosphatase 1) (PHPT1)	PHP14	0.03	1.03 ↑
P08134	Rho-related GTP-binding protein RhoC (Rho cDNA clone 9) (h9)	RHOC	0.03	1.01 ↑
P26599	Polypyrimidine tract-binding protein 1 (PTB) (57 kDa RNA-binding protein PPTB-1) (Heterogeneous nuclear ribonucleoprotein I) (hnRNP I)	PTBP1	0.03	1.01 ↑
P63104	14-3-3 protein zeta/delta (Protein kinase C inhibitor protein 1) (KCIP-1)	1433Z	0.03	1.01 ↑
P11586	C-1-tetrahydrofolate synthase, cytoplasmic (C1-THF synthase) (Epididymis secretory sperm binding protein)	C1TC	0.03	1.01 ↑
P55735	Protein SEC13 homolog (GATOR complex protein SEC13) (SEC13-like protein 1) (SEC13-related protein)	SEC13	0.03	1.01 ↑
Q8TAT6	Nuclear protein localization protein 4 homolog (Protein NPL4)	NPL4	0.03	1.02 ↑
Q9Y230	RuvB-like 2 (48 kDa TATA box-binding protein-interacting protein)	RUVB2	0.03	1.01 ↑
P11216	Glycogen phosphorylase, brain form	PYGB	0.03	1.02 ↑
Q99613	Eukaryotic translation initiation factor 3 subunit C (eIF3c)	EIF3C	0.03	1.01 ↑
Q13151	Heterogeneous nuclear ribonucleoprotein A0 (hnRNP A0)	ROA0	0.03	1.02 ↑
P17980	26S proteasome regulatory subunit 6A (26S proteasome AAA-ATPase subunit RPT5)	PRS6A	0.03	1.02 ↑
Q9UQ80	Proliferation-associated protein 2G4 (Cell cycle protein p38-2G4 homolog) (hG4-1) (ErbB3-binding protein 1)	PA2G4	0.03	1.01 ↑
P62979	Ubiquitin-ribosomal protein eS31 fusion protein (Ubiquitin carboxyl extension protein 80)	RS27A	0.03	1.02 ↑
Q9UMX0	Ubiquilin-1 (Protein linking IAP with cytoskeleton 1) (PLIC-1) (hPLIC-1)	UBQL1	0.03	1.03 ↑
Q9UQ35	Serine/arginine repetitive matrix protein 2 (300 kDa nuclear matrix antigen)	SRRM2	0.03	1.07 ↑
Q9Y266	Nuclear migration protein nudC (Nuclear distribution protein C homolog)	NUDC	0.02	1.03 ↑

Protein IDs	Protein Names	Gene IDs	*p*-value	Fold Change
Q9Y5B9	FACT complex subunit SPT16 (Chromatin-specific transcription elongation factor 140 kDa subunit)	SP16H	0.02	1.01 ↑
Q8TEX9	Importin-4 (Imp4) (Importin-4b) (Imp4b) (Ran-binding protein 4) (RanBP4)	IPO4	0.02	1.06 ↑
Q9UNZ2	NSFL1 cofactor p47 (UBX domain-containing protein 2C) (p97 cofactor p47)	NSF1C	0.02	1.10 ↑
Q99426	Tubulin-folding cofactor B (Cytoskeleton-associated protein 1)	TBCB	0.02	1.02 ↑
P62753	Small ribosomal subunit protein eS6 (40S ribosomal protein S6) (Phosphoprotein NP33)	RS6	0.02	1.15 ↑
P09211	Glutathione S-transferase P (GST class-pi) (GSTP1-1)	GSTP1	0.02	1.01 ↑
Q71U36	Tubulin alpha-1A chain (Alpha-tubulin 3) (Tubulin B-alpha-1) (Tubulin alpha-3 chain)	TBA1A	0.02	1.01 ↑
Q96T88	E3 ubiquitin-protein ligase UHRF1 (Inverted CCAAT box-binding protein of 90 kDa) (Nuclear protein 95)	UHRF1	0.02	1.03 ↑
P62917	Large ribosomal subunit protein uL2 (60S ribosomal protein L8)	RL8	0.02	1.04 ↑
P15880	Small ribosomal subunit protein uS5	RS2	0.02	1.02 ↑
P35579	Myosin-9 (Cellular myosin heavy chain, type A)	MYH9	0.02	1.01 ↑
P15559	NAD(P)H dehydrogenase [quinone] 1	NQO1	0.02	1.01 ↑
P62888	Large ribosomal subunit protein eL30 (60S ribosomal protein L30)	RL30	0.02	1.01 ↑
Q14247	Src substrate cortactin (Amplaxin) (Oncogene EMS1)	SRC8	0.02	1.03 ↑
Q9BRA2	Thioredoxin domain-containing protein 17 (14 kDa thioredoxin-related protein) (TRP14)	TXD17	0.02	1.03 ↑
P22234	Bifunctional phosphoribosylaminoimidazole carboxylase/phosphoribosylaminoimidazole succinocarboxamide synthetase (PAICS)	PUR6	0.02	1.01 ↑
P0DMV9	Heat shock 70 kDa protein 1B (Heat shock 70 kDa protein 2) (HSP70-2) (HSP70.2)	HS71B	0.02	1.01 ↑
Q9UKV3	Apoptotic chromatin condensation inducer in the nucleus (Acinus)	ACINU	0.02	1.05 ↑
Q8WWM7	Ataxin-2-like protein (Ataxin-2 domain protein) (Ataxin-2-related protein)	ATX2L	0.02	1.06 ↑
Q99829	Copine-1 (Chromobindin 17) (Copine I)	CPNE1	0.02	1.02 ↑
P50395	Rab GDP dissociation inhibitor beta (Rab GDI beta) (Guanosine diphosphate dissociation inhibitor 2) (GDI-2)	GDIB	0.02	1.01 ↑

Protein IDs	Protein Names	Gene IDs	*p*-value	Fold Change
P23528	Cofilin-1 (18 kDa phosphoprotein) (p18) (Cofilin, non-muscle isoform)	COF1	0.02	1.01 ↑
O75396	Vesicle-trafficking protein SEC22b (ER-Golgi SNARE of 24 kDa)	SC22B	0.02	1.04 ↑
Q08J23	RNA cytosine C(5)-methyltransferase NSUN2	NSUN2	0.02	1.05 ↑
Q96FW1	Ubiquitin thioesterase OTUB1	OTUB1	0.02	1.03 ↑
P23526	Adenosylhomocysteinase (AdoHcyase)	SAHH	0.02	1.02 ↑
Q9BWM7	Sideroflexin-3	SFXN3	0.02	1.02 ↑
Q9BVG4	Protein PBDC1 (Polysaccharide biosynthesis domain-containing protein 1)	PBDC1	0.02	1.06 ↑
Q99497	Parkinson disease protein 7 (Maillard deglycase) (Oncogene DJ1) (Parkinsonism-associated deglycase) (Protein DJ-1) (DJ-1) (Protein/nucleic acid deglycase DJ-1)	PARK7	0.02	1.01 ↑
P11940	Polyadenylate-binding protein 1 (PABP-1) (Poly(A)-binding protein 1)	PABP1	0.02	1.02 ↑
P27708	CAD protein [Includes: Glutamine-dependent carbamoyl-phosphate synthase	PYR1	0.02	1.01 ↑
P05386	Large ribosomal subunit protein P1 (60S acidic ribosomal protein P1)	RLA1	0.02	1.02 ↑
P30419	Glycylpeptide N-tetradecanoyltransferase 1	NMT1	0.02	1.02 ↑
P16989	Y-box-binding protein 3 (Cold shock domain-containing protein A)	YBOX3	0.01	1.03 ↑
P36578	Large ribosomal subunit protein uL4 (60S ribosomal protein L1)	RL4	0.01	1.03 ↑
O00299	Chloride intracellular channel protein 1 (Chloride channel ABP)	CLIC1	0.01	1.01 ↑
Q15365	Poly(rC)-binding protein 1 (Alpha-CP1)	PCBP1	0.01	1.02 ↑
Q99536	Synaptic vesicle membrane protein VAT-1 homolog	VAT1	0.01	1.01 ↑
P62269	Small ribosomal subunit protein uS13 (40S ribosomal protein S18) (Ke-3) (Ke3)	RS18	0.01	1.01 ↑
Q15785	Mitochondrial import receptor subunit TOM34 (hTom34)	TOM34	0.01	1.01 ↑
P33316	Deoxyuridine 5'-triphosphate nucleotidohydrolase, mitochondrial (dUTPase)	DUT	0.01	1.02 ↑
P50995	Annexin A11 (56 kDa autoantigen) (Annexin XI)	ANX11	0.01	1.03 ↑
Q14204	Cytoplasmic dynein 1 heavy chain 1	DYHC1	0.01	1.01 ↑
P30041	Peroxiredoxin-6	PRDX6	0.01	1.01 ↑
P41567	Eukaryotic translation initiation factor 1 (eIF1)	EIF1	0.01	1.11 ↑

Protein IDs	Protein Names	Gene IDs	*p*-value	Fold Change
P39019	Small ribosomal subunit protein eS19 (40S ribosomal protein S19)	RS19	0.01	1.01 ↑
P26038	Moesin (Membrane-organizing extension spike protein)	MOES	0.01	1.02 ↑
A0A0U1RRL7	Protein MMP24OS (MMP24 opposite strand)	MMPOS	0.01	1.04 ↑
Q16186	Proteasomal ubiquitin receptor ADRM1 (110 kDa cell membrane glycoprotein) (Gp110)	ADRM1	0.01	1.04 ↑
P11413	Glucose-6-phosphate 1-dehydrogenase (G6PD)	G6PD	0.01	1.02 ↑
Q92530	Proteasome inhibitor PI31 subunit (hPI31)	PSMF1	0.01	1.05 ↑
P50914	Large ribosomal subunit protein eL14 (60S ribosomal protein L14) (CAG-ISL 7)	RL14	0.01	1.02 ↑
P08758	Annexin A5 (Anchorin CII) (Annexin V)	ANXA5	0.01	1.02 ↑
P21333	Filamin-A (FLN-A)	FLNA	0.01	1.01 ↑
Q92900	Regulator of nonsense transcripts 1	RENT1	0.009	1.02 ↑
Q14011	Cold-inducible RNA-binding protein (A18 hnRNP) (Glycine-rich RNA-binding protein CIRP)	CIRBP	0.009	1.02 ↑
O95197	Reticulon-3 (Homolog of ASY protein) (HAP) (Neuroendocrine-specific protein-like 2) (NSP-like protein 2) (Neuroendocrine-specific protein-like II) (NSP-like protein II) (NSPLII)	RTN3	0.008	1.04 ↑
O43175	D-3-phosphoglycerate dehydrogenase (3-PGDH)	SERA	0.008	1.03 ↑
P56537	Eukaryotic translation initiation factor 6 (eIF-6)	IF6	0.008	1.02 ↑
P23381	Tryptophan--tRNA ligase, cytoplasmic (Interferon-induced protein 53) (IFP53)	SYWC	0.007	1.04 ↑
O43765	Small glutamine-rich tetratricopeptide repeat-containing protein alpha (Alpha-SGT)	SGTA	0.007	1.03 ↑
O14744	Protein arginine N-methyltransferase 5 (PRMT5)	ANM5	0.007	1.02 ↑
Q9NTK5	Obg-like ATPase 1 (DNA damage-regulated overexpressed in cancer 45) (DOC45) (GTP-binding protein 9)	OLA1	0.007	1.03 ↑
P46087	Probable 28S rRNA (cytosine(4447)-C(5))-methyltransferase	NOP2	0.007	1.03 ↑
P68366	Tubulin alpha-4A chain	TBA4A	0.007	1.04 ↑
Q9UJU6	Drebrin-like protein (Cervical SH3P7)	DBNL	0.006	1.04 ↑
Q16658	Fascin (55 kDa actin-bundling protein) (Singed-like protein) (p55)	FSCN1	0.006	1.01 ↑
P62241	Small ribosomal subunit protein eS8 (40S ribosomal protein S8)	RS8	0.006	1.02 ↑
P50570	Dynamin-2	DYN2	0.005	1.03 ↑
P11021	Endoplasmic reticulum chaperone BiP	BIP	0.005	1.01 ↑

Protein IDs	Protein Names	Gene IDs	*p*-value	Fold Change
Q14019	Coactosin-like protein	COTL1	0.005	1.02 ↑
P31150	Rab GDP dissociation inhibitor alpha (Rab GDI alpha)	GDIA	0.005	1.02 ↑
P10599	Thioredoxin (Trx) (ATL-derived factor) (ADF)	THIO	0.005	1.01 ↑
P15311	Ezrin (Cytovillin) (Villin-2) (p81)	EZRI	0.005	1.01 ↑
P49591	Serine--tRNA ligase, cytoplasmic	SYSC	0.005	1.04 ↑
P30043	Flavin reductase (NADPH) (FR)	BLVRB	0.005	1.02 ↑
P13639	Elongation factor 2 (EF-2)	EF2	0.004	1.01 ↑
P16949	Stathmin (Leukemia-associated phosphoprotein p18)	STMN1	0.004	1.07 ↑
P46781	Small ribosomal subunit protein uS4 (40S ribosomal protein S9)	RS9	0.004	1.02 ↑
P41250	Glycine--tRNA ligase (Diadenosine tetraphosphate synthetase) (Ap4A synthetase)	GARS	0.004	1.01 ↑
Q9BWF3	RNA-binding protein 4 (Lark homolog) (hLark) (RNA-binding motif protein 4) (RNA-binding motif protein 4a)	RBM4	0.004	1.02 ↑
P30086	Phosphatidylethanolamine-binding protein 1 (PEBP-1)	PEBP1	0.004	1.02 ↑
Q07955	Serine/arginine-rich splicing factor 1 (Alternative-splicing factor 1)	SRSF1	0.004	1.02 ↑
P08195	4F2 cell-surface antigen heavy chain (4F2hc)	4F2	0.004	1.02 ↑
P17655	Calpain-2 catalytic subunit (Calcium-activated neutral proteinase 2) (CANP 2)	CAN2	0.004	1.03 ↑
P14618	Pyruvate kinase PKM(Cytosolic thyroid hormone-binding protein) (CTHBP)	KPYM	0.003	1.01 ↑
Q01081	Splicing factor U2AF 35 kDa subunit (U2 auxiliary factor 35 kDa subunit)	U2AF1	0.003	1.04 ↑
P30085	UMP-CMP kinase (Deoxycytidylate kinase) (CK) (dCMP kinase)	KCY	0.003	1.01 ↑
P05387	Large ribosomal subunit protein P2 (60S acidic ribosomal protein P2) (Renal carcinoma antigen NY-REN-44)	RLA2	0.003	1.02 ↑
P62829	Large ribosomal subunit protein uL14 (60S ribosomal protein L17) (60S ribosomal protein L23)	RL23	0.003	1.05 ↑
P13489	Ribonuclease inhibitor (Placental ribonuclease inhibitor) (Placental RNase inhibitor) (Ribonuclease/angiogenin inhibitor 1) (RAI)	RINI	0.002	1.02 ↑
P04083	Annexin A1 (Annexin I)	ANXA1	0.002	1.02 ↑
P24534	Elongation factor 1-beta (EF-1-beta)	EF1B	0.002	1.02 ↑

Protein IDs	Protein Names	Gene IDs	*p*-value	Fold Change
P42677	Small ribosomal subunit protein eS27 (40S ribosomal protein S27) (Metallopan-stimulin 1) (MPS-1)	RS27	0.002	1.06 ↑
Q92945	Far upstream element-binding protein 2 (FUSE-binding protein 2) (KH type-splicing regulatory protein) (KSRP) (p75)	FUBP2	0.002	1.01 ↑
Q15942	Zyxin (Zyxin-2)	ZYX	0.001	1.10 ↑
P12268	Inosine-5'-monophosphate dehydrogenase 2 (IMP dehydrogenase 2) (IMPD 2)	IMDH2	0.001	1.03 ↑
P19105	Myosin regulatory light chain 12A (Epididymis secretory protein Li 24) (HEL-S-24)	ML12A	0.001	1.01 ↑
P67809	Y-box-binding protein 1 (YB-1) (CCAAT-binding transcription factor I subunit A) (CBF-A)	YBOX1	0.0004	1.04 ↑
Q13501	Sequestosome-1 (EBI3-associated protein of 60 kDa) (EBIAP) (p60)	SQSTM	0.0004	1.08 ↑
P62913	Large ribosomal subunit protein uL5 (60S ribosomal protein L11)	RL11	0.0003	1.02 ↑
P54577	Tyrosine--tRNA ligase, cytoplasmic (TyrRS)	SYYC	0.0003	1.03 ↑
P62266	Small ribosomal subunit protein uS12 (40S ribosomal protein S23)	RS23	0.0002	1.11 ↑
P31946	14-3-3 protein beta/alpha (Protein 1054) (Protein kinase C inhibitor protein 1) (KCIP-1)	1433B	0.0002	1.01 ↑
P40222	Alpha-taxilin	TXLNA	0.0002	1.04 ↑
P63244	Small ribosomal subunit protein RACK1 (Cell proliferation-inducing gene 21 protein)	RACK1	0.0001	1.01 ↑
Q9H1E3	Nuclear ubiquitous casein and cyclin-dependent kinase substrate 1 (P1)	NUCKS	0.00009	1.04 ↑
P24666	Low molecular weight phosphotyrosine protein phosphatase (LMW-PTP)	PPAC	0.00005	1.03 ↑
P51858	Hepatoma-derived growth factor (HDGF) (High mobility group protein 1-like 2) (HMG-1L2)	HDGF	0.00005	1.02 ↑
Q16543	Hsp90 co-chaperone Cdc37 (Hsp90 chaperone protein kinase-targeting subunit) (p50Cdc37)	CDC37	0.00003	1.03 ↑
P26641	Elongation factor 1-gamma (EF-1-gamma)	EF1G	0.00002	1.03 ↑
